# SHMT2 and the BRCC36/BRISC deubiquitinase regulate HIV-1 Tat K63-ubiquitylation and destruction by autophagy

**DOI:** 10.1371/journal.ppat.1007071

**Published:** 2018-05-23

**Authors:** Muyu Xu, James J. Moresco, Max Chang, Amey Mukim, Davey Smith, Jolene K. Diedrich, John R. Yates, Katherine A. Jones

**Affiliations:** 1 Regulatory Biology Laboratory, The Salk Institute for Biological Studies, La Jolla, CA, United States of America; 2 Mass Spectrometry Core for Proteomics and Metabolomics, The Salk Institute for Biological Studies, La Jolla, CA, United States of America; 3 Razavi Newman Integrative Genomics and Bioinformatics Core, The Salk Institute for Biological Studies, La Jolla, CA, United States of America; 4 Division of Infectious Diseases, University of California San Diego School of Medicine, La Jolla, CA, United States of America; 5 Department of Molecular Medicine, Scripps Research Institute, La Jolla, CA, United States of America; Duke University Medical Center, UNITED STATES

## Abstract

HIV-1 Tat is a key regulator of viral transcription, however little is known about the mechanisms that control its turnover in T cells. Here we use a novel proteomics technique, called DiffPOP, to identify the molecular target of JIB-04, a small molecule compound that potently and selectively blocks HIV-1 Tat expression, transactivation, and virus replication in T cell lines. Mass-spectrometry analysis of whole-cell extracts from 2D10 Jurkat T cells revealed that JIB-04 targets Serine Hydroxymethyltransferase 2 (SHMT2), a regulator of glycine biosynthesis and an adaptor for the BRCC36 K63Ub-specific deubiquitinase in the BRISC complex. Importantly, knockdown of SHMT1,2 or BRCC36, or exposure of cells to JIB-04, strongly increased Tat K63Ub-dependent destruction via autophagy. Moreover, point mutation of multiple lysines in Tat, or knockdown of BRCC36 or SHMT1,2, was sufficient to prevent destruction of Tat by JIB-04. We conclude that HIV-1 Tat levels are regulated through K63Ub-selective autophagy mediated through SHMT1,2 and the BRCC36 deubiquitinase.

## Author summary

The HIV-1 Tat protein is critical for virus expression and replication, but little is known about the factors that control its expression level in T cells. Here, we have investigated a small molecule compound, JIB-04, which induces the rapid and selective destruction of the HIV-1 Tat protein in HeLa and T cells, with minimal effects on host cell transcription. To better understand the regulation of Tat stability, we adopted a new proteomics approach (DiffPOP) designed to identify drug-protein targets based on their differential solubility in the presence of the drug. Through this process, we identified the SHMT2 enzyme, a regulator of glycine biosynthesis and an adaptor for the BRCC36 K63-specific deubiquitinase, as a prominent molecular target of JIB-04. We show that turnover of Tat by JIB-04 is accompanied by a large increase in Tat K63-Ub, but not Tat K48-Ub, and that destruction occurs via selective autophagy. Importantly, mutation of eight lysine residues in Tat, or knockdown of BRCC36 or SHMT1,2, was sufficient to prevent destruction of Tat by JIB-04. Taken together, these findings identify SHMT2 and BRCC36 as novel and important regulators of HIV-1 Tat protein levels in infected T cells.

## Introduction

The HIV-1 Tat transactivator stimulates RNAPII transcription elongation at the proviral promoter in response to increased levels of the positive transcription elongation factor-b (P-TEFb/CDK9) complex in activated T cells [1–3]. The expression of early viral transcripts encoding Tat effectively establishes a positive feedback loop that up-regulates viral late genes and enables virion production. In the absence of this supportive environment, the integrated HIV-1 provirus can persist for long periods of time in a latent but fully competent state [4]. Because Tat protein levels are limiting in latently-infected resting memory CD4+ T cells, events that facilitate the assembly and activation of functional Tat:P-TEFb complexes are generally sufficient to counteract viral latency [5–7]. Thus, Tat-mediated induction of HIV-1 transcription leads to full-blown infection and virus replication in activated T cells. For these reasons, it is important to fully define the mechanism of Tat transactivation, and identify the factors that control Tat expression levels in infected cells.

Previous studies have established that Tat stimulates transcription elongation at the viral promoter by activating and recruiting the P-TEFb/CDK9 kinase to the TAR RNA hairpin structure in the 5’-LTR [1–4]. P-TEFb alleviates RNAPII pausing by phosphorylating and releasing the negative elongation factor, NELF, from the nascent TAR RNA [8]. The Tat:P-TEFb complex also phosphorylates the Spt5 elongation factor, which is then able to interact with additional elongation factors, and recruits the Super Elongation Complex [9]. Our lab further showed that Tat binds and activates the RNAPII C-terminal domain (CTD) phosphatase, Ssu72, which removes CTD-Ser5 phosphorylation (Ser5P) from paused RNAPII complexes [10]. Removal of RNAPII CTD-Ser5P is necessary for the Tat:P-TEFb complex to access and phosphorylate the RNAPII CTD at the Ser2 and Ser7 positions in the heptad repeats, which can then attract splicing and histone modification factors required for mRNA processing. Lastly, Tat can also stimulate mRNA 5’-end capping, through direct interaction with the capping enyzmes [11]. Together, these data suggest a model in which one or more molecules of Tat function at the HIV-1 promoter and promoter-proximal TAR RNA to couple mRNA 5’-end capping with the recruitment of SSU-72 and P-TEFb, facilitate the RNAPII CTD Ser5P to Ser7P transition, and phosphorylate elongation factors to release the paused RNAPII elongation complex.

Compared to our detailed understanding of the Tat transactivation process, relatively little is known about the factors that control Tat expression and proteolytic turnover in infected cells. Because intracellular Tat levels are tightly controlled in infected cells and strongly increased by T cell signaling [12], the enzymes that control Tat turnover are likely to also be important regulators of HIV-1 replication and latency. Previous studies have shown that Tat is modified by site-specific acetylation, methylation and ubiquitylation, which affect its transactivation activity and binding to TAR RNA [13–19]. Tat stability is determined in part by K48Ub and proteolytic degradation through the proteasome [20]. However, other studies have highlighted a prominent role for selective autophagy in the control of Tat protein levels [21,22]. Consistent with these findings, autophagy has been shown to be a potent virus restriction factor [23,24]. To date, little is known about the details of Tat destruction through autophagy. Although proteins or misfolded protein aggregates destined for autophagy are often modified by high levels of K63-ubiquitylation (K63Ub) [25–27], the interaction of Tat with the autophagy factor SQSTM1/p62 was found to be independent of ubiquitin [21], raising the question of whether ubiquitin has a significant role in the control of Tat stability, and which factors act as adaptors for Tat lysosomal proteolysis and destruction.

Here we report that HIV-1 Tat protein levels in HeLa and T cells are strongly affected by the histone demethylase inhibitor, JIB-04 [28]. Unexpectedly, knockdown of putative histone demethylase targets did not recapitulate the effect of JIB-04 on Tat expression. To better understand how JIB-04 controls Tat stability, we used a novel mass-spectrometry approach, called DiffPOP, which can identify protein:drug conjugates via their differential solubility in methanol. Through this approach, we identified the serine hydroxymethyltransferase enzyme, SHMT2, as a direct target of JIB-04 in Jurkat 2D10 T cells. SHMT2, and the related SHMT1 protein, are metabolic enzymes required for nuclear thymidylate biosynthesis during S phase [29]. In addition, SHMT2 was recently shown to deliver substrates to the cytoplasmic BRCC36/BRISC complex specifically for removal of K63 ubiquitylation [30,31]. In this latter role, SHMT2 was found to stabilize the type 1 interferon (IFN) receptor chain 1 (IFNAR1) in interferon-signaling cells [32]. In particular, the removal of K63Ub by BRCC36 was sufficient to protect IFNAR1 from lysosomal degradation during autophagy. Here we show that SHMT1,2 and the BRCC36/BRISC deubiquitase complex are novel and selective regulators of Tat K63, but not K48, ubiquitylation, and its degradation through selective autophagy in T cells.

## Results

### JIB-04 inhibits HIV-1 Tat expression and transactivation

**Fig 1:**
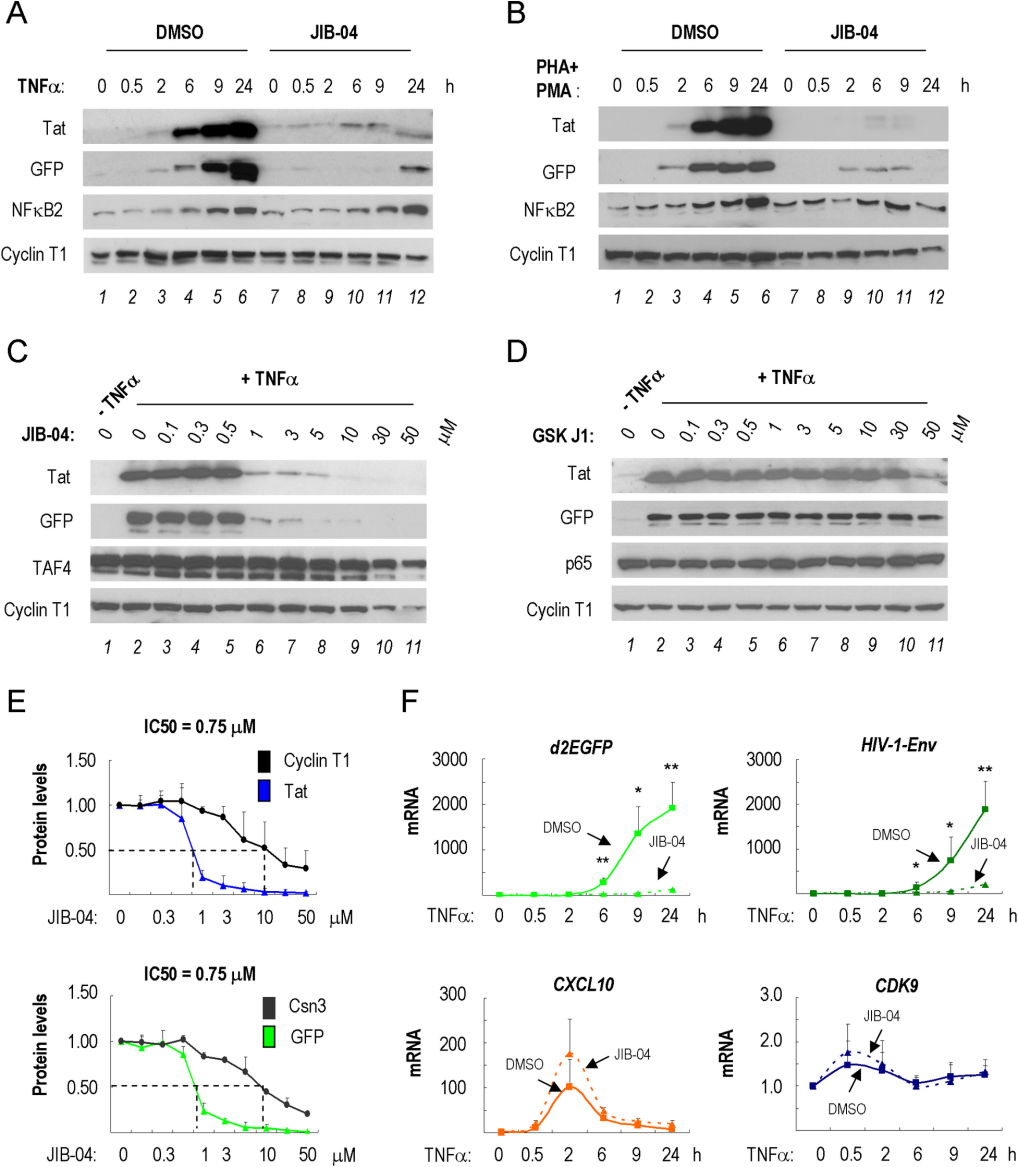
JIB-04 inhibits HIV-1 Tat expression in activated 2D10 T cells. (**A**) Immunoblot analysis of HIV-1 Tat expression from the integrated HIV-1 provirus in 2D10 T cells induced with TNFα for 0–24 h, as indicated above each lane. Cells were treated with DMSO (lanes 1–6) or 3 μM JIB-04 (lanes 7–12) overnight. Cyclin T1 served as loading control. (**B**) As in part A, except that 2D10 cells were activated with PHA (10 μg/mL) and PMA (50 ng/mL) for the times indicated above each lane. (**C**) Immunoblot analysis of HIV-1 Tat protein levels in 2D10 cells treated with different concentrations of JIB-04 (0.1 μM-50 μM) for 24 h, as indicated. Cyclin T1 and TAF4 (TFIID subunit) served as loading controls. (**D**) As in part C, except that cells were exposed to the histone demethylase inhibitor GSKJ1, at the concentrations listed above each lane. Cyclin T1 and p65 (NF-κB subunit) served as loading controls. (**E**) Quantification of Tat and eGFP protein levels in TNFα-stimulated 2D10 cells treated with the JIB-04 at the concentrations indicated in the X-axis. The signals were calculated by Image J and averaged from three independent experiments. (**F**) qRT-PCR analysis *d2EGFP* (HIV-1 reporter gene), *HIV-1 Env*, or host cell *CXCL10* and *CDK9* mRNAs extracted from 2D10 cells pre-treated with either DMSO or 3 μM JIB-04 for 16 h and stimulated by TNFα (10 ng/ml), for the times indicated below each graph. Values on the Y-axis for *d2EGFP*, *Env*, *CXCL10* and *CDK9* mRNAs prior to TNFα stimulation were normalized to 1. Differences between DMSO and 3 μM-JIB-04 treated samples were calculated by Student’s T-test at each time point (*p<0.05, **p<0.005, ***p<0.0005). Shown is a representative result from three independent experiments.

Based on reports that HIV-1 is regulated by histone demethylases [33,34], we examined the effect of histone demethylase inhibitors on HIV-1 Tat transactivation in 2D10 T cells, a Jurkat-derived cell line containing a single integrated HIV-1 provirus reporter gene [5]. Among several compounds tested (JIB-04, GSKJ1, ML324, IOX1 and 2-PCPA), only the pyridine hydrazine JIB-04 [28] strongly blocked Tat expression at low concentrations (Fig 1A and S1D Fig). Importantly, JIB-04 also potently blocked expression of the d2EGFP reporter gene inserted into the viral Nef open reading frame in this cell line (S1A Fig). Parallel experiments revealed that JIB-04 similarly suppressed induction of Tat in response to PMA/PHA in 2D10 cells (Fig 1B), indicating that the effect of this compound is not stimulus or pathway-specific. Further analysis revealed that JIB-04 blocked Tat expression in a dose-dependent manner (Fig 1C). By contrast, Tat expression in activated 2D10 cells was unaffected by a different histone demethylase inhibitor, GSKJ1 (only a minor reduction at 50 μM, Fig 1D). Detailed analysis of the effects of JIB-04 on Tat and eGFP protein expression established an approximate IC50 of 0.75 μM in 2D10 T cells (Fig 1E). Exposure of TNFα-treated 2D10 T cells to JIB-04 (3 μM) strongly blocked expression of HIV-1-encoded Env and d2EGFP mRNAs (Fig 1F and S1C Fig). By contrast, JIB-04 had no effect on the level of CDK9 mRNA, or induction of the CXCL10 gene by TNFα. Together, these findings suggest that JIB-04 strongly blocks Tat expression and transactivation of the integrated HIV-1 LTR in 2D10 T cells.

**Fig 2:**
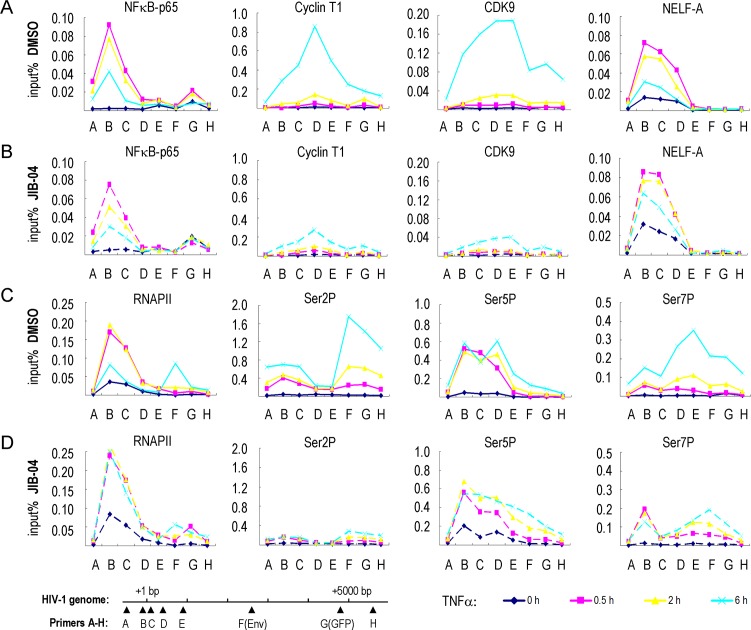
ChIP analysis of the effect of JIB-04 on the binding of transcription factors to the HIV-1 genome. ChIP analysis comparison of transcription factor binding to the single integrated HIV-1 genome in 2D10 T cells treated either with DMSO (A,C) or with 3 μM JIB-04 (B,D). Cells were treated with TNFα (10 ng/ml) for 0 h (blue line), 0.5 h (pink line), 2 h (yellow line), or 6 h (light blue line). The ChIP values in the Y-axis are expressed as percentage input. The ChIP primers (A-G) that were used are indicated on the X-axis, and their relative location on the HIV-1 genome are shown in the schematic at the bottom left. Antisera used for ChIP antibodies are NF-κB (p65 subunit), Cyclin T1, CDK9, NELF-A, RNAPII CTD, and phosphorylated RNAPII CTD-Ser2 (Ser2P), CTD-Ser5 (Ser5P) and CTD-Ser7 (Ser7P), as indicated above each panel.

To assess how JIB-04 affects binding of transcription factors to the HIV-1 promoter, we carried out ChIP experiments at the integrated HIV-1 genome in 2D10 cells in the presence and absence of the drug (Fig 2 and S2 Fig). As expected, TNFα treatment led to the rapid recruitment of NF-κB and RNAPII (Fig 2A and 2C, pink line), whereas P-TEFb (CyclinT1 and CDK9 panels) occupancy was observed only at later times (Fig 2A, light blue line), following T cell activation and accumulation of Tat. As we showed previously [10], the binding of NF-κB and RNAPII is accompanied by recruitment of the NELF negative elongation factor to establish the paused RNAPII complex (Fig 2A, pink line), and NELF is displaced at later times upon binding of Tat and P-TEFb (light blue line). JIB-04 did not affect the binding of NF-κB (compare Fig 2A and 2B, pink line). Levels of total RNAPII at the HIV-1 promoter did not decrease following TNFα stimulation in JIB-04 treated cells, as they do in DMSO-treated cells, indicating a block to elongation (compare Fig 2C and 2D, light blue line). Consistent with the rapid loss of Tat expression, JIB-04 treatment dramatically reduced P-TEFb occupancy at the HIV-1 promoter (Fig 2B, light blue line). Consistent with this possibility, dramatically reduced levels of elongation-competent RNAPII CTD-Ser2P and -Ser7P were observed within the coding region of the provirus in JIB-04 treated cells (compare Fig 2C and 2D, light blue line). Furthermore, the release of NELF-A from HIV-1 promoter did not occur in JIB-04 treated cells (compare Fig 2A and 2B, light blue line). Taken together, these data show that JIB-04 strongly blocks Tat-dependent transcription elongation in vivo.

**Fig 3:**
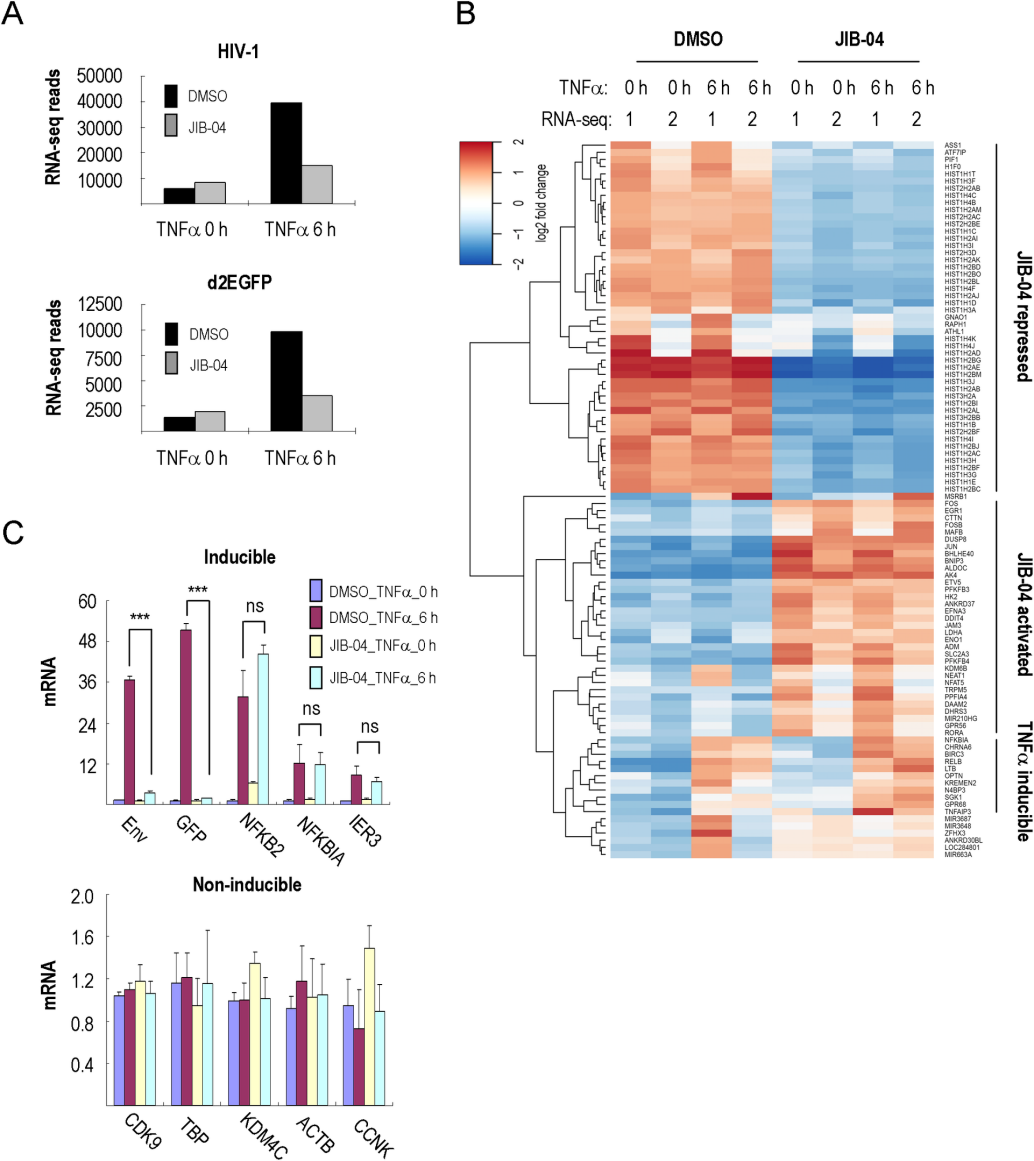
JIB-04 has a limited effect on host cell transcription. (**A**) Shown is a graph of normalized RNA-seq reads mapped to HIV-1 and eGFP in 2D10 cells. Cells were pre-treated with DMSO or 3 μM JIB-04 for 16 h, and stimulated by TNFα (10 ng/ml) for 0h or 6h, as indicated. (**B**) RNA-seq heatmap of 100 genes that showed the largest differences in expression in DMSO versus JIB-04-treated 2D10 cells. The effect of JIB-04 on host cell gene expression was monitored both in the absence (0h) or presence (6h) of TNFα. Data were derived from minimum and the maximum rlog values of duplicate RNA-seq experiments in 2D10 cells. Genes are grouped as JIB-04-repressed, JIB-04-activated and TNFα-inducible groups, as indicated on the right. (**C**) Analysis of mRNA by qRT-PCR for *HIV-Env*, *eGFP* and selected TNFα-inducible (top) and non-inducible (bottom) genes in 2D10 cells in DMSO or JIB-04-treated cells. Significant differences between samples treated by DMSO or 3 μM JIB-04 at 6 h TNFα stimulation were calculated by Student’s T-test (*p<0.05, **p<0.005, ***p<0.0005; ns = non-significant) for each gene.

These observations raised the question of whether JIB-04 inhibits histone demethylase enzymes required for HIV-1 transcription, or whether it can directly or indirectly inhibit P-TEFb similar to compounds like flavopiridol, which inhibits host cell transcription and induces a strong host cell stress response [35,36]. To assess this question, 2D10 cells were treated with JIB-04 in the presence or absence of TNFα, and total RNA was extracted for analysis by next-generation RNA sequencing (RNA-seq). As expected, the reads mapping to the HIV-1 genome and to eGFP were dramatically reduced in JIB-04 treated cells upon activation by TNFα (Fig 3A), consistent with the loss of Tat protein expression in these cells. By contrast, the effect of JIB-04 on host cell mRNA levels was quite modest. Overall, JIB-04 affected the expression of only 811 genes by more than 2-fold (p<0.05), out of 13546 genes monitored. Of the JIB-04-affected genes, only 413 (51%) were up-regulated and 398 (49%) were down-regulated (S3A Fig and S1 File). Gene ontology analysis indicated that the most avidly repressed host cell genes included canonical histone genes, and that many of the genes activated by JIB-04 were associated with glycolysis (Fig 3B, S3B and S3C Fig). Despite these changes at the mRNA level, JIB-04 had limited effect on global histone protein levels in 2D10 cells (S3D Fig). Moreover, JIB-04 did not affect the expression of TNFα-inducible genes (*NF-κB2*, *NF-κBIA*, *IER3* and *CD83*; Fig 3B, 3C and S1 File), consistent with the ChIP data showing that the drug does not affect the binding of NF-κB. The RNA-seq results were validated by qRT-PCR analysis of select genes in JIB-04 treated 2D10 cells (Fig 3C and S3C Fig). Taken together, these findings indicate that JIB-04 does not inhibit transcription broadly, nor does it affect signaling through the NF-κB pathway.

**Fig 4:**
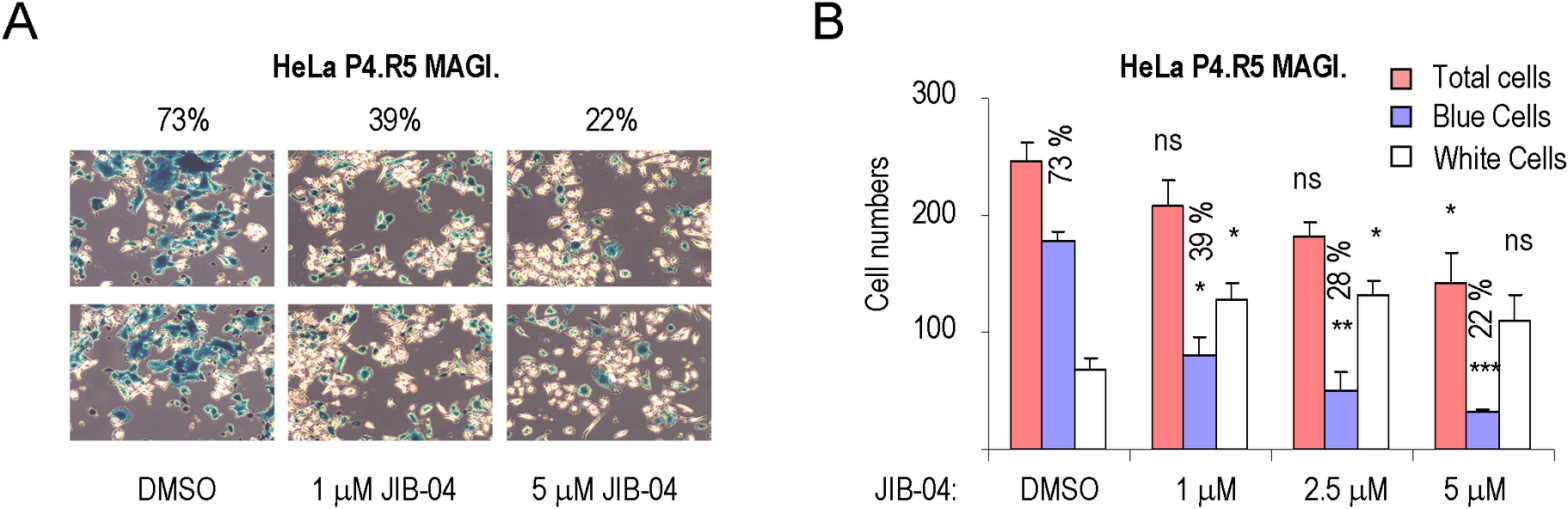
JIB-04 inhibits HIV replication in HeLa P4.R5 MAGI. indicator cells. (**A**) Images of two representative fields from single-cycle infectivity imaging assays for HeLa P4.R5 MAGI cells subjected to HIV-1 infection and treated with either DMSO or JIB-04. The numbers above each panel refer to the percentage of HIV-infected blue cells exposed to DMSO, 1 μM JIB-04, or 5 μM JIB-04. Shown are two representative photos taken from each of three replicate plates, with a 10X10 amplification. (**B**) Graph plotting the average numbers of total (pink bar), blue (blue bar) or white (white bar) cells from at least three representative photos in the presence of DMSO or different concentrations of JIB-04 (1–5 μM). The percentage HIV-infected (blue) cells is indicated above the blue bars. Significant differences between cell numbers treated by DMSO or different concentrations of JIB-04 for total, blue and white cells were calculated by Student’s T-test (*p<0.05, **p<0.005, ***p<0.0005; ns = non-significant), respectively.

We next tested whether JIB-04 blocks virus replication in the HeLa P4.R5 MAGI. indicator cell line. This cell line contains an integrated β-galactosidase reporter under the control of the HIV-1 LTR promoter, and infected cells can be visualized by staining with an X-gal substrate, which turns blue in the presence of β-galactosidase. For this experiment, we analyzed JIB-04 at concentrations (1–5 μM) that did not strongly affect cell viability. Untreated cells or cells treated with JIB-04 were challenged with active Nef-deleted HIV-1 (pNL4-3), as described previously [37]. As shown in Fig 4A and 4B, JIB-04 significantly reduced the percentage of HIV-infected blue cells (from 73% to 22%) with little effect on HeLa P4.R5 MAGI. cell viability, indicating the compound strongly inhibits virus replication.

We next analyzed the effect of JIB-04 in primary CD4+ T cells isolated from peripheral blood from a healthy donor. Primary CD4+ T cells were infected with wild-type NL4-3 virus and treated with DMSO or JIB-04 at various concentrations (S4 Fig). Although JIB-04 efficiently inhibited HIV replication, we noted that cell viability decreased sharply, indicating that the compound is toxic to primary T cells. As expected, the integrase inhibitor Raltegravir blocked HIV-1 replication in this assay, without affecting cell viability. Taken together, these data indicated that JIB-04 is a potent inhibitor of Tat expression and activity in cell lines, but is not suitable for analysis in primary T cells.

### Mass spectrometry (DiffPOP) identification of SHMT2 as a direct target of JIB-04

**Fig 5:**
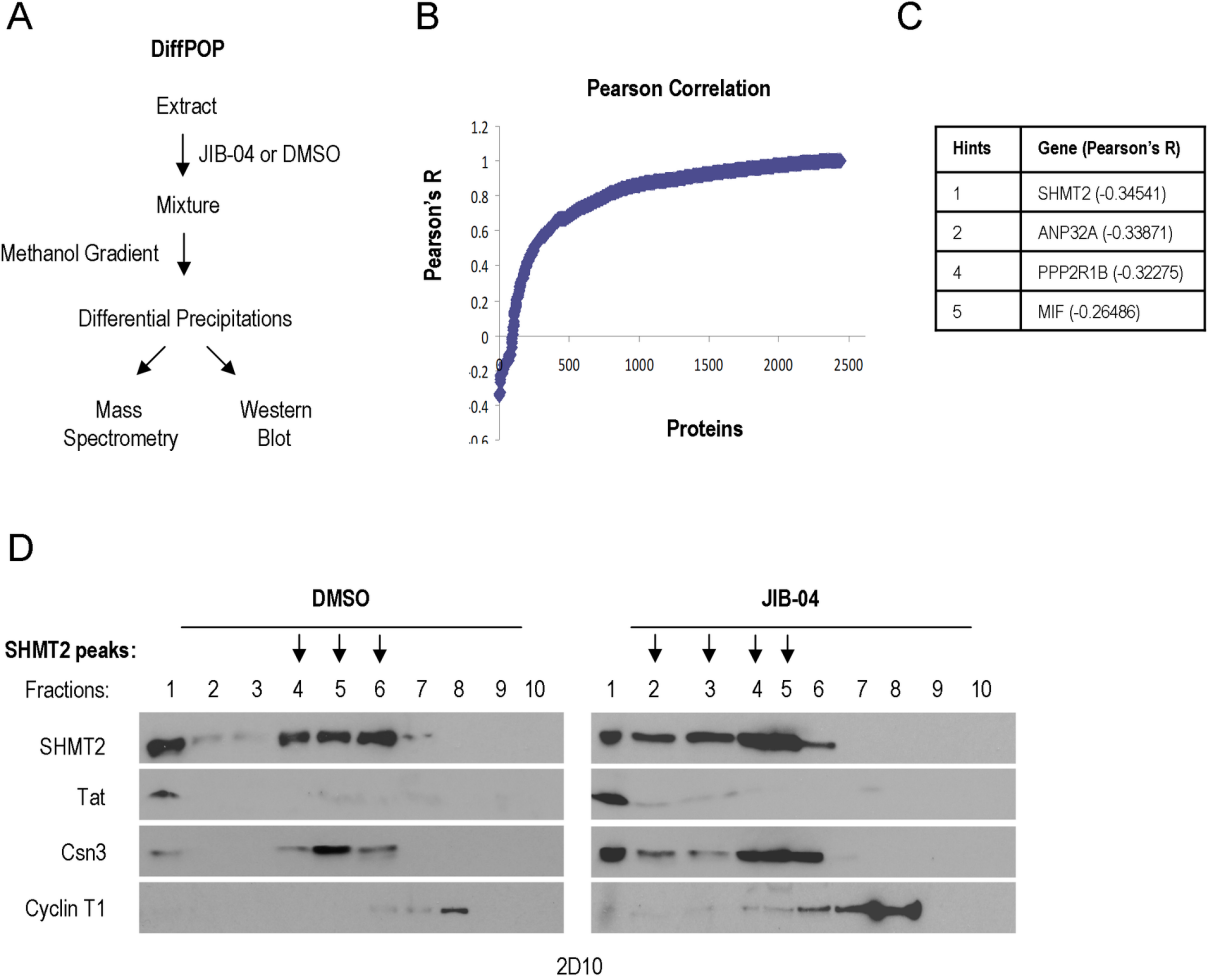
DiffPOP analysis identifies the SHMT2 serine hydroxymethyltransferase as a target of JIB-04. (**A**) Schematic diagram of the DiffPOP protocol. Soluble proteins in 2D10 whole cell extracts were precipitated in fractions containing increasing concentrations of methanol, in the presence and absence of the JIB-04 compound. The protein composition of the different precipitates was then assessed using mass-spectrometry proteomics analysis. (**B**) Pearson Correlation coefficients were calculated to identify factors that show altered protein solubility in methanol when exposed to DMSO or 200 μM JIB-04. (**C**) The top five candidate host cell proteins targeted by JIB-04, defined as those with the smallest Pearson’s R value. The complete protein list is shown in S2 File. (**D**) Manual verification of DiffPOP results for SHMT2. Extracts precipitated at different methanol concentrations in the presence or absence of JIB-04 were analyzed by immunoblot for endogenous SHMT2. The results also show that the methanol solubility of HIV-1 Tat, Csn3, and Cyclin T1 proteins did not change in response to JIB-04. The peak of SHMT2 shifted from fractions 4–6 in the DMSO-treated fractions to fractions 2–5 in the extracts exposed to JIB-04.

JIB-04 has been shown previously to inhibit various histone demethylases (KDM5A, KDM4A-E, KDM6B; 28). However, knockdown of individual histone demethylases, including various KDM4s, KDM5A, 5B and JMJD6 failed to recapitulate the effects of JIB-04 on Tat expression and activity in TNFα-treated 2D10 and HeLa cells (S5 Fig). Based on these findings, and the observation that JIB-04 does not broadly affect host cell transcription or CDK9/P-TEFb expression (Fig 3), we sought to identify additional targets for JIB-04 in 2D10 cells. To achieve this goal, we adapted a technique currently in development at The Salk Mass Spectrometry Core and Yates laboratory (TSRI, La Jolla, CA), to identify drug interaction targets in an unbiased manner. This approach, termed DiffPOP, is based on the differential solubility of drug:protein conjugates as compared to unconjugated proteins in the presence of methanol gradients. In brief, 2D10 whole-cell extracts were treated with either DMSO or JIB-04, and the soluble proteins were precipitated in a stepwise fashion in fractions containing increasing concentrations of methanol (Fig 5A). Proteins present in the precipitates of each fraction were then identified using mass-spectrometry.

This approach established that JIB-04 has no effect on the solubility of the vast majority of proteins in the 2D10 cell extracts. However, a few proteins were found to precipitate differently in extracts treated with JIB-04, as determined by analysis of the Pearson’s correlation coefficient (Fig 5B and S2 File). The top host cell protein target identified by this technique was the host cell serine hydroxymethyltransferase enzyme, SHMT2 (Fig 5C). SHMT2, and the related SHMT1 enzyme, are multifunctional proteins that regulates one-carbon metabolism and nucleotide biosynthesis. Relevant to our study, SHMT2 was also shown to stabilize the interferon receptor (IFNAR1), by targeting it to the cytosolic BRCC36 K63-specific deubiquitinase complex. The results of the DiffPOP analysis were verified manually by immunoblot analysis of 2D10 extract protein fractions precipitated at different methanol concentrations (Fig 5D). In the presence of JIB-04, the fractionation profile of SHMT2 was altered (indicated with arrows), whereas the solubility of HIV-1 Tat, and host cell factors Csn3 and Cyclin T1 was unchanged (peaks at fractions 1, 5 and 8, respectively). Taken together, these data strongly suggest that JIB-04 binds SHMT2 and alters its solubility in methanol.

### JIB-04 promotes Tat protein ubiquitination and degradation

**Fig 6:**
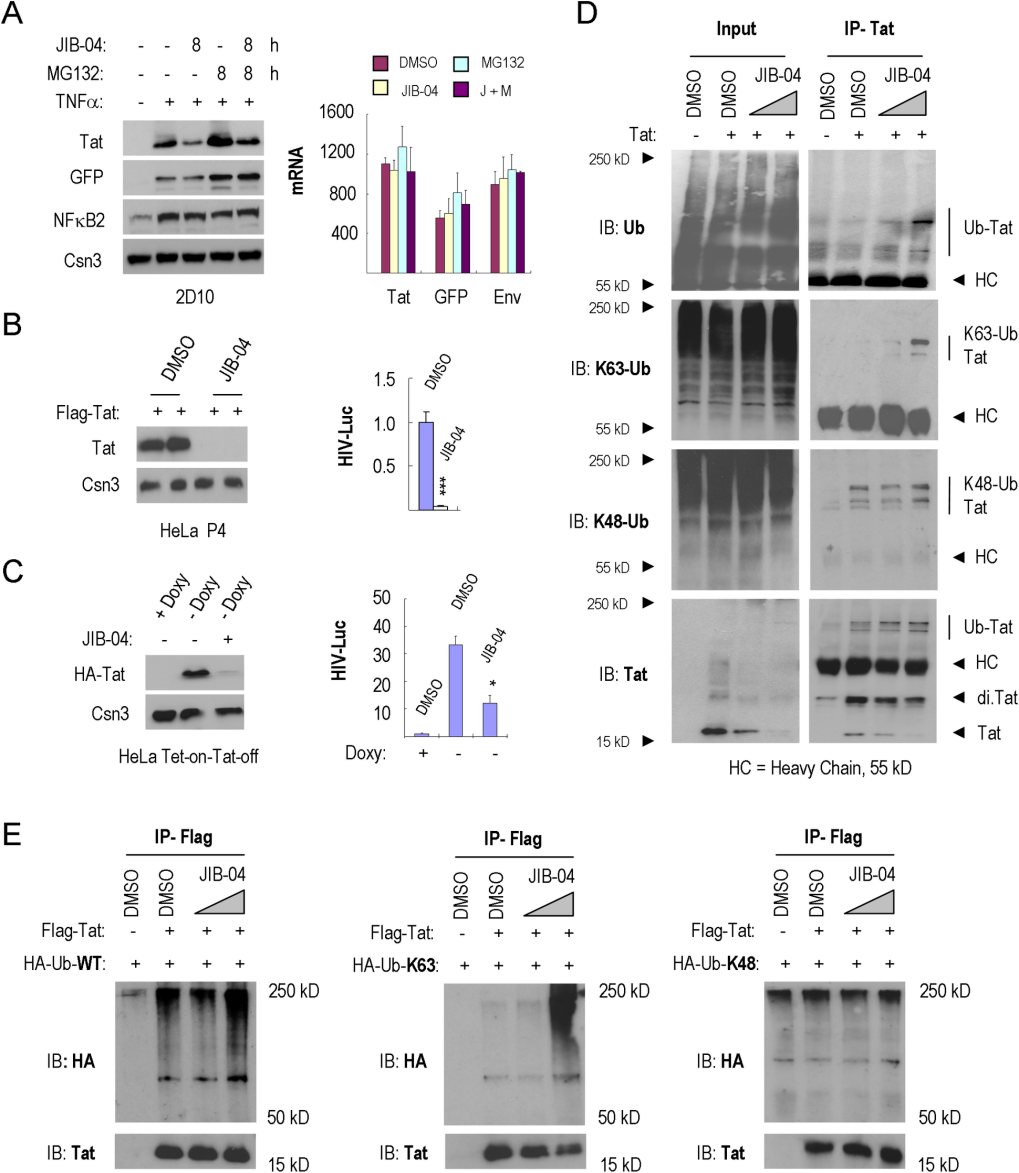
JIB-04 increases Tat K63Ub and proteolytic destruction. (**A**) Left, immunoblot analysis of HIV-1 Tat expression in 2D10 cells exposed to DMSO, JIB-04, MG132 or JIB-04+MG132. Cells were pre-treated with or without TNFα (10 ng/ml) for 16 h. The COP9 signalosome complex subunit 3 (Csn3) served as loading control. Right, qRT-PCR analysis of *Tat*, *GFP* and *Env* mRNA levels in these cells. Values shown in the Y-axis were normalized to mRNAs from 2D10 cells without TNFα-stimulation. (**B**) Dual-Luc (HIV-LTR-Luc/SV40-Renilla-Luc) reporter gene analysis in FLAG-Tat101 transfected HeLa P4 cells. Left, immunoblot analysis of FLAG-Tat101 protein levels in cells treated with DMSO or 2 μM JIB-04. Right panels show dual-luc reporter gene activity in these cells. Significant differences between HIV-Luc activity treated by DMSO or 2 μM JIB-04 were calculated by Student’s T-test (*p<0.05, **p<0.005, ***p<0.0005). (**C**) Dual-Luc reporter gene analysis, as in part B, in Tet-on-Tat-off HeLa cells. Left, immunoblot analysis of HA-Tat86 protein levels in 2D10 cells treated by DMSO or 2.5 μM JIB-04. Right, dual-Luc reporter gene activity in these cells. Significant differences between HIV-Luc activity treated by DMSO or 2.5 μM JIB-04 were calculated by Student’s T-test (*p<0.05, **p<0.005, ***p<0.0005). (**D**) Analysis of the effect of JIB-04 on endogenous Tat K63Ub levels in HeLa cells. HIV-1 Tat was immunoprecipitated from lysates of HeLa cells exposed to DMSO or JIB-04 (1 μM and 3 μM), and endogenous ubiquitylation was monitored using the antisera indicated to the left of each panel (HC = antibody heavy chain). (**E**) Immunoprecipitation of FLAG-Tat-101 from lysates of HeLa cells treated with DMSO or JIB-04 (1μM and 3μM). Ubiquitination of FLAG-Tat-101 in the presence of ectopically expressed HA-ubiquitin-WT, HA-ubiquitin-K63-only or HA-ubiquitin-K48-only was assessed using anti-HA antisera.

These findings raised the question of whether SHMT2 regulates Tat turnover, potentially by serving to target Tat to the BRCC36/BRISC K63-specific deubiquitinase (DUB) complex, as observed for IFNAR1 in interferon-treated cells [31]. The BRCC36 deubiquitinase is a subunit of both nuclear (BRCA1-A) and cytoplasmic (BRISC) complexes. Nuclear BRCA1-A complexes have been implicated in histone deubiquitylation during DNA repair [38], whereas cytosolic BRISC complexes deubiquitylates and stabilizes various substrate proteins. Consistent with this possibility, we noted that JIB-04 affected HIV-1 Tat protein levels at early timepoints, prior to the decline of Tat mRNA or eGFP protein levels (Fig 6A). Of note, each of these proteins has a short half-life of less than two hours (S6C Fig). We next asked whether JIB-04 would also control Tat protein levels in stably expressed cell lines. Interesting, incubation with JIB-04 markedly reduced FLAG-Tat expression in HeLa P4 cells, as well as HA-Tat levels in a Tet-on-Tat-off HeLa cell line (Fig 6B and 6C). JIB-04 also inhibited the activity of the HIV-Luciferase reporter gene in each cell line, without affecting the activity of SV40:Luciferase or CMV:β-galactosidase reporter genes (Fig 6B, 6C and S6B Fig). Moreover, JIB-04 reduced Tat protein levels, but not mRNA levels, in a dose-dependent manner (S6A Fig). Taken together, these data show that the primary effect of JIB-04 is at the level of protein Tat stability, rather than transcription. Consistent with this possibility, measurements in the presence of cycloheximide revealed that the half-life of the Tat strongly decreased in response to JIB-04 exposure in 2D10 T cells (S6C Fig).

We next asked whether JIB-04 affects HIV-1 Tat ubiquitylation. Interestingly, immunoprecipitation of Tat protein under stringent conditions revealed a dose-dependent increase in total ubiquitylation in response to JIB-04 in HeLa Tet-on-Tat-off cells (Fig 6D). Immunoblot analysis using K48Ub- and K63Ub-specific antisera revealed that JIB-04 selectively increased endogenous Tat-K63Ub, but not Tat-K48Ub, levels. Similarly, JIB-04 increased the fraction of Flag-Tat protein modified by HA-ubiquitin in HeLa P4 cells (Fig 6E). Transfection of HA-tagged ubiquitin mutants that only allow either K63 or K48 linkages further established that JIB-04 selectively increased FLAG-Tat K63, but not K48, ubiquitylation. Taken together, these data indicates that JIB-04 exposure strongly increases HIV-1 Tat K63Ub and proteolytic turnover.

**Fig 7:**
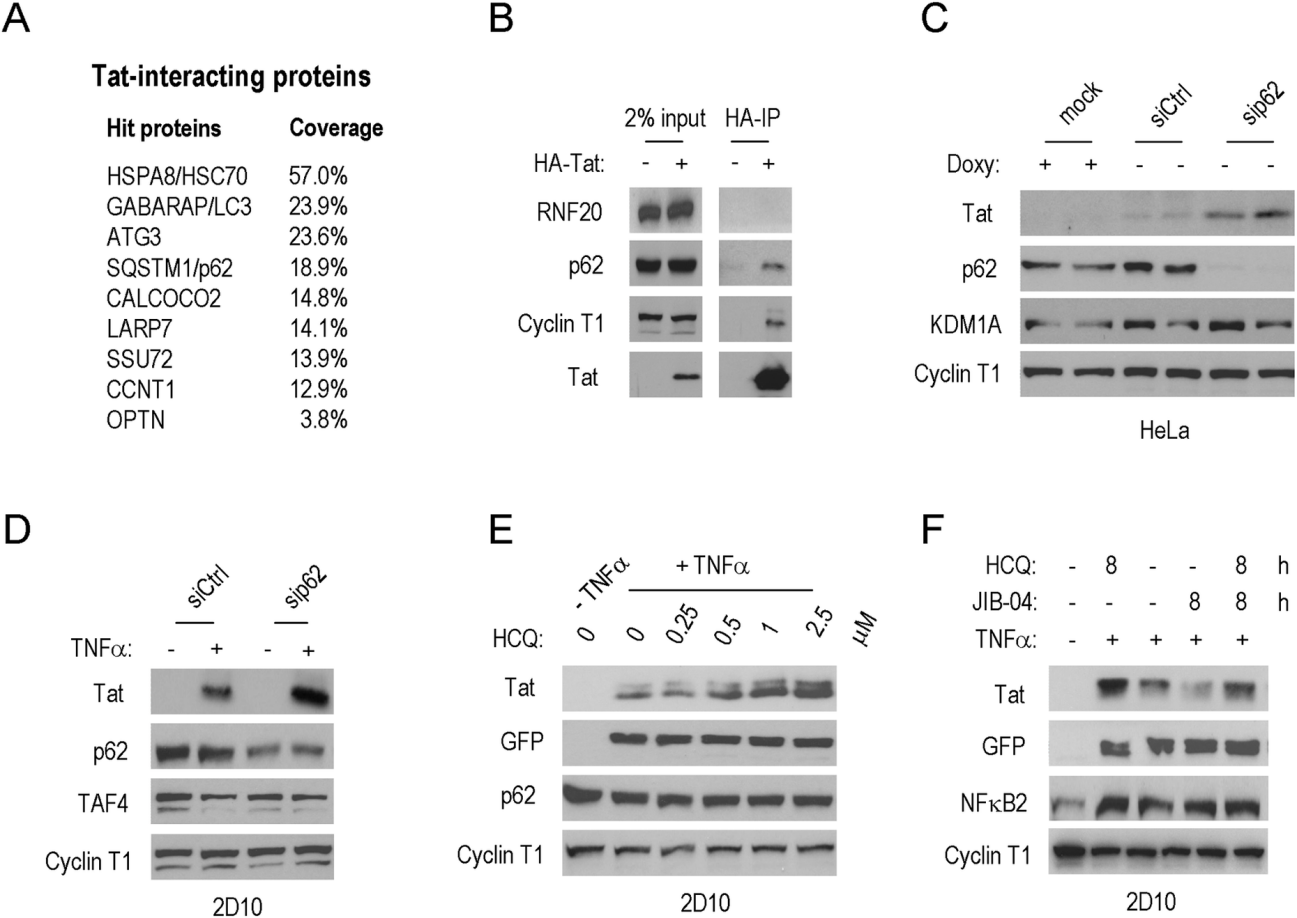
JIB-04 promotes destruction of HIV-1 Tat by selective autophagy. (**A**) MudPIT proteomics analysis of HA-Tat immunoaffinity purified complexes in HeLa cells. The table lists factors present in HA-Tat complexes, ranked by sequence coverage. The list includes several autophagy regulators, as well as well-known Tat-associated proteins (Cyclin T1, SSU72). (**B**) Immunoblot analysis to confirm the association of SQSMT1/p62 with HIV-1 Tat. (**C**) Immunoblot of Tat protein levels in HeLa cells depleted of the autophagy regulator SQSTM1/p62. Cyclin T1 served as loading control. (**D**) Immunoblot analysis of Tat protein levels in TNFα-stimulated 2D10 cells depleted of SQSTM1/p62. Cyclin T1 served as loading control. (**E**) Immunoblot analysis of HIV-1 Tat protein levels in 2D10 cells exposed to an inhibitor of lysosomal proteases, hydroxychloroquine (HCQ). Cyclin T1 served as loading control. (**F**) Immunoblot analysis of the effect of JIB-04 on Tat protein levels in 2D10 cells treated with the autophagy inhibitor HCQ. Cyclin T1 served as loading control.

Previous studies have established that whereas K48Ub predisposes proteins to degradation through the proteasome, K63Ub of protein aggregates and inclusion bodies targets proteins for proteolytic destruction via autophagy [25,26]. Consistent with a prevalent role for autophagy in the destruction of Tat, our proteomics analysis of HA-Tat complexes by MudPIT (Multi-dimensional Protein Identification Technology) revealed the presence of several autophagy regulators, including HSC70, LC3, ATG3, and SQSTM1/p62 (Fig 7A). An interaction with SQSTM1/p62 was further confirmed by co-immunoprecipitation with HA-Tat (Fig 7B), and knockdown of SQSTM1/p62 in HeLa or 2D10 cells modestly increased Tat protein levels (Fig 7C and 7D). Moreover, treatment of 2D10 cells with the autophagy inhibitor, hydroxychloroquine (HCQ) [39], strongly increased Tat protein levels in a dose-dependent manner (Fig 7E and S7A Fig), whereas we did not observe stabilization by 3-Methyladenine (3-MA; S7B Fig). Lastly, cells treated with JIB-04 failed to degrade Tat in cells treated with the autophagy inhibitor, HCQ (Fig 7F). These data link the effects of JIB-04 to the destruction of Tat through the process of selective autophagy.

### SHMT2 and the BRCC36/BRISC deubiquitinase regulate Tat-K63Ub and turnover

**Fig 8:**
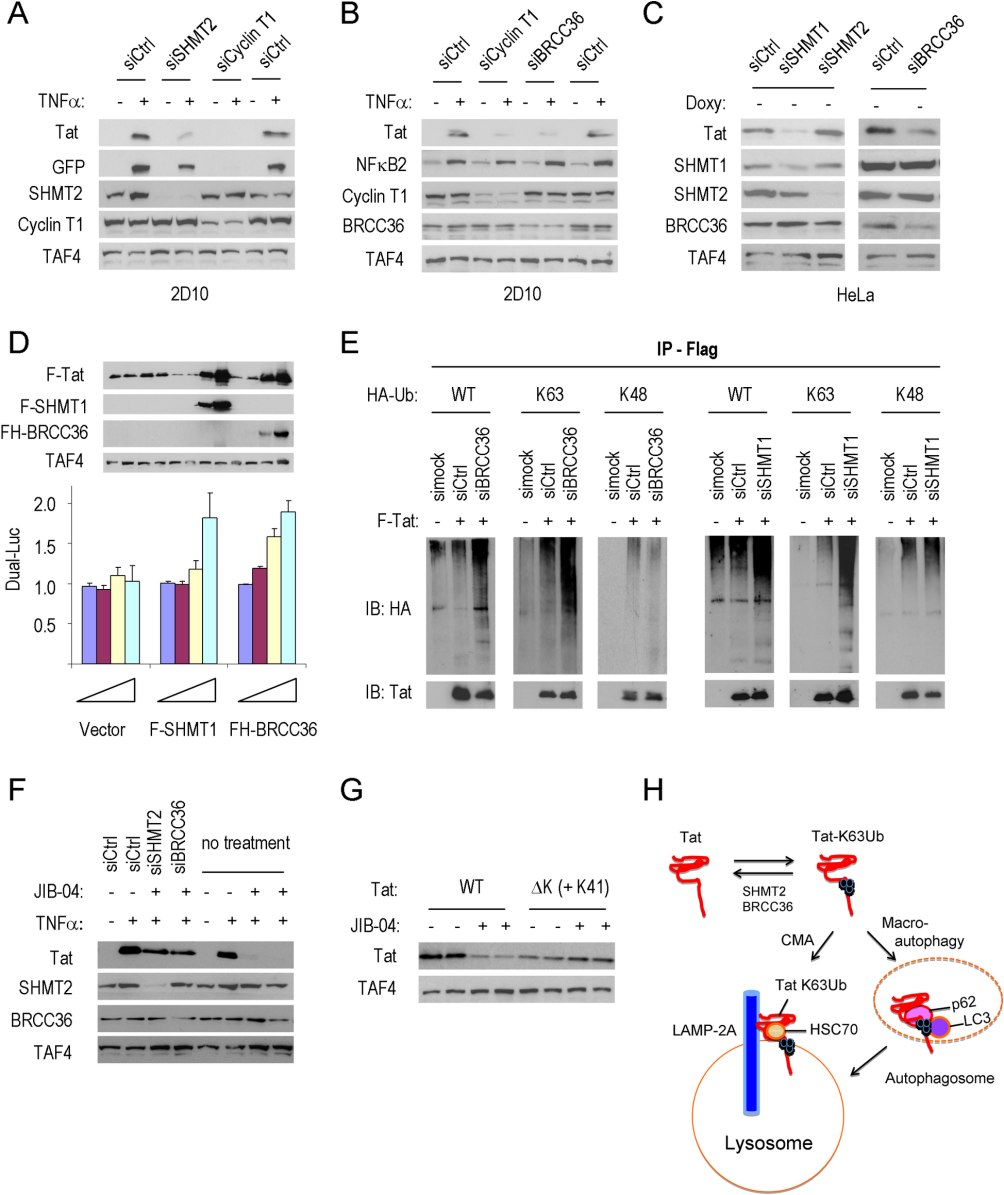
SHMT2 and the BRCC36/BRISC deubiquitinase controls Tat K63Ub and destruction by autophagy. (**A**) Immunoblot analysis of Tat and GFP protein levels in 2D10 cells depleted of SHMT2 or Cyclin T1, as indicated. TAF4 served as loading control. (**B**) Immunoblot analysis of Tat protein expression in 2D10 cells depleted of BRCC36 or Cyclin T1. NF-κB2 protein levels were monitored to assess any change in T cell signaling. TAF4 served as loading control. (**C**) Immunoblot analysis of Tat expression in Tet-on-Tat-off HeLa cells depleted of SHMT1, SHMT2 or BRCC36, as indicated above each lane. TAF4 served as loading control. (**D**) Top, immunoblot analysis of the effects of overexpression of Flag-SHMT1 and FH-BRCC36 proteins on Tat protein levels. TAF4 served as loading control. Bottom, Dual:Luc reporter activity of the HIV-Luc reporter. Plasmids expressing the Flag-vector, Flag-SHMT1 and FH-BRCC36 proteins were tested at 0 ng (blue bar), 20 ng (purple bar), 100 ng (yellow bar) and 500 ng (light blue bar), as indicated. (**E**) Analysis of FLAG-Tat-101 immunoprecipitates from lysates of BRCC36- or SHMT1- knockdown cells. FLAG-Tat-101 proteins were labelled with HA-tagged ubiquitin, either wild-type Ub (WT), or ubiquitin mutants that selectively support only K63 or K48 ubiquitylation, and Tat proteins were monitored using anti-HA antisera. (**F**) Immunoblot analysis of the effect of JIB-04 on Tat protein levels in 2D10 cells depleted of SHMT2 or BRCC36. (**G**) The effect of JIB-04 on wildtype or Tat ΔK (+K41) STREP-tagged Tat proteins was shown by immunoblot. TAF4 served as loading control. (**H**) Model of the role of SHMT2 and BRCC36 in the release of Tat-K63Ub from destruction through chaperone-mediated autophagy or SQSMT1/p62-dependent macroautophagy.

We next analyzed whether SHMT2 and the BRCC36/BRISC K63Ub deubiquitinase control Tat K63Ub levels and stability in 2D10 cells. Importantly, knockdown of SHMT2 strongly reduced Tat protein levels as early as 24h following after siRNA transfection in 2D10 cells (Fig 8A and S8A Fig). Similarly, knockdown of the BRCC36 K63Ub-specific deubiquitinase [31, 32, 40] led to a drastic decline in Tat protein levels in 2D10 or HeLa cells (Fig 8B and 8C). Additional knockdown experiments revealed that Tat stability is controlled by SHMT1, rather than SHMT2, in HeLa cells (Fig 8C). Consequently, the residual Tat expression in 2D10 cells depleted of SHMT2 might be due to redundant actions of SHMT1. Further analysis showed that knockdown of SHMT1,2 or BRCC36 reduced Tat protein stability without affecting mRNA levels (S8A, S8B and S8C Fig). Analysis of the Tat protein half-life in cycloheximide-treated cells revealed a sharp reduction in SHMT2-depleted 2D10 cells (S8D Fig), indicating that SHMT2 controls Tat stability. Additionally, JIB-04 did not affect Tat levels in cells depleted of either SHMT2 or BRCC36, confirming that both enzymes are required for the response to the drug (Fig 8F). Moreover, ectopic expression of FLAG-SHMT1 or FH-BRCC36 strongly increased FLAG-Tat protein levels and HIV-Luc activity in HeLa cells (Fig 8D). Importantly, depletion of either BRCC36 or SHMT1 in HeLa cells also selectively increased K63Ub, but not K48Ub, of the FLAG-Tat protein (Fig 8E). Collectively, these data strongly indicate that JIB-04 promotes Tat destruction by interfering with SHMT2 and the BRCC36 deubiquitinase.

It was previously shown that single lysine mutations were not sufficient to eliminate Tat-K63Ub in infected cells [19], and similarly, we found that individual lysine point mutations were not sufficient to stabilize Tat in JIB-04-treated cells (S8E and S8F Fig). Whereas the Tat ΔK mutant (all lysines substituted by arginine) was not stable in HeLa cells, the Tat ΔK (+K41) mutant, with eight lysines replaced with arginine [19], was relatively well expressed (S8G Fig). Most importantly, JIB-04 did not affect the stability of the Tat ΔK (+K41) mutant protein (Fig 8G). These findings establish that multiple lysine residues in Tat are required for turnover in response to JIB-04. Taken together, these data suggested a model through which SHMT2 and BRCC36 act to reverse K63Ub of Tat, thereby preventing its destruction by autophagy (Fig 8H).

## Discussion

In this study, we show that HIV-1 Tat protein is marked by K63Ub for selective autophagy and coupled lysosomal destruction. This study began with the fortuitous discovery that the histone demethylase (HDM) inhibitor JIB-04 markedly down-regulates Tat protein levels and HIV-1 LTR transactivation in T cell lines. The JIB-04 compound strongly decreased Tat protein stability in 2D10 cells, where native Tat is expressed from the HIV-1 genome, as well as in other cell lines where tagged Tat proteins are expressed from heterologous promoters. ChIP experiments at the integrated HIV-1 LTR confirmed a selective block to RNAPII transcription elongation in cells treated with JIB-04. RNA-seq studies further established that despite the dramatic effect of JIB-04 on viral transcripts, the compound had only limited effects on host cell transcription and NF-κB signaling. Thus JIB-04 acts differently from compounds like flavopiridol, which inhibits the P-TEFb elongation factor [35, 41] and affects host cell transcription broadly.

Although JIB-04 is known to inhibit histone demethylases (HDM) [28], knockdown of multiple HDMs did not recapitulate the effect of the drug on Tat stability. To identify other factors that may be sensitive to JIB-04, we adapted a novel mass-spectrometry technique, called DiffPOP, which identifies proteins based on their altered solubility in methanol in the presence of a drug. Using this approach, we identified SHMT2 (serine hydroxymethyltransferase-2) as a novel target of JIB-04 in 2D10 cell extracts. Importantly, knockdown of SHMT2 in 2D10 cells, or the related SHMT1 protein in HeLa cells, strongly decreased Tat protein expression, without affecting mRNA levels. Thus, depletion of SHMT2 was able to recapitulate the effects of JIB-04 on Tat protein levels, and validated the results of the DiffPOP approach. Of note, the histone demethylases targeted by JIB-04 were not detected by the DiffPOP approach because they remained soluble in the highest levels of methanol tested in our experiments. Because SHMT2, and the related SHMT1 enzyme, supplies methyl groups for one-carbon pools used for nucleotide biosynthesis and histone and DNA methylation [42–44], JIB-04 might also modulate histone methylation indirectly, through inhibition of SHMT2.

In addition to its role in one-carbon metabolism, SHMT2 was previously shown to stabilize the IFNAR1 receptor by targeting it to the cytosolic BRCC36 K63-specific deubiquitinase (DUB) complex in interferon-treated cells [31]. The BRCC36 deubiquitinase is a subunit of distinct nuclear (BRCA1-A) and cytoplasmic (BRISC) complexes. Nuclear BRCA1-A complexes are predominantly involved in histone deubiquitylation during DNA repair [38], whereas cytosolic BRCC36/BRISC complexes deubiquitylate and stabilize protein substrates, including the IFNAR1 interferon receptor [31], the inflammasome component NLRP3 [45] and components of the mitotic spindle [46]. Importantly, knockdown of BRCC36, like SHMT2, led to a dramatic loss of Tat protein levels, concomitant with a sharp increase of Tat-K63, but not -K48, ubiquitylation. These findings strongly implicate K63 ubiquitylation of Tat as a mechanism to target it for selective destruction through autophagy, as has been seen with other autophagy substrates [25,26]. Consistent with these findings, we show that Tat protein levels increase dramatically in cells with the autophagy inhibitor hydroxychloroquine, which permeabilizes lysosomal membranes, blocking both macroautophagy as well as chaperone-mediated autophagy. Taken together, these data strongly suggest that SHMT2 and BRCC36/BRISC cooperate to rescue Tat from destruction through removal of K63Ub. Importantly, previous studies have shown that SHMT2 levels are massively upregulated upon T cell activation [47]. These findings indicate that T cell activation not only increases Tat protein biosynthesis, through upregulation of Cyclin T1 protein and enhanced HIV-1 LTR transactivation, but also decreases Tat turnover due to increased expression of SHMT2 and removal of Tat-K63Ub.

Previous studies have shown that Tat K48Ub can stimulate transactivation without affecting Tat turnover [14, 19]. However, the potential role of Tat K63Ub in its destruction has been unclear, in part because mutation of single lysine residues does not increase Tat protein stability, and because Tat does not bind the SQSMT1/p62 autophagy factor through the ubiquitin-interaction domain [21]. Several lines of evidence indicate that Tat turnover by autophagy is dependent on K63Ub. First, we show that increased Tat turnover in cells exposed to JIB-04, or in cells depleted of SHMT2 or BRCC36, is accompanied by a large increase in Tat-K63Ub levels, with no effect on Tat K48Ub. Because BRCC36 is capable of removing only K63Ub chains, it does not require specific targeting of substrates through the K63Ub modification. Second, we show that point mutation of multiple lysine residues in Tat is sufficient to stabilize protein levels, and renders Tat protein levels insensitive to proteolysis induced by JIB-04. Third, the residual levels of Tat that remain following depletion of either SHMT2 or BRCC36 are not sensitive to JIB-04. Taken together, these data provide strong evidence that Tat levels are regulated by selective autophagy through a K63Ub-dependent mechanism.

Selective protein destruction can occur either through canonical macroautophagy or through chaperone-mediated autophagy (CMA), or both, however the mechanisms of each pathway are quite distinct [48, 49]. For CMA, client proteins interact through a KFERQ-like motif with HSC70 chaperones and are targeted directly to the LAMP2A lysosomal membrane receptor for destruction by proteases in the lysosomal lumen. For macroautophagy, K63Ub client proteins interact with the ubiquitin-binding domain of SQSMT1/p62. Previous studies have shown that Tat interacts with p62 and LC3 for destruction by macroautophagy [21]. However, Tat proteins in the cytoplasm have also been found to strongly co-localize with the LAMP2A lysosome receptor in neuronal cells [50]. Our proteomics analysis of Tat complexes reveals high levels of the CMA-specific chaperone, HSC70, in addition to the macroautophagy proteins p62 and LC3, which suggests that Tat may be destroyed through either process, depending on the specific environment of the cell. Therefore further studies will be needed to distinguish the relative contributions of canonical (macroautophagy) and non-canonical (CMA) pathways to Tat turnover during latency and active infection, where both pathways are known to be functional [51], and to assess whether or not SHMT2 and BRCC36 function selectively in one pathway.

In summary, the data presented here show that Tat-K63Ub proteins can be rescued from autophagy through the combined actions of SHMT2 and the BRCC36 deubuiquitinase, which may be important for the rapid induction of HIV-1 transcription upon T cell activation. Different parameters that affect Tat half-life have important considerations for establishment and escape from viral latency [7]. Further examination of the mechanisms that control Tat K63Ub levels and its in turnover by autophagy may enable a more robust induction of Tat expression for escape from latency to potentiate the effectiveness of antiviral regimens.

## Methods

### Commercial antisera used in this study

Specific antisera were obtained from the following sources: HIV-1 Tat (ab42359 & ab43014), GFP (sc-9996), HA (Sigma, H9658-.2ML, clone HA-7 & Novus, NB600363), Flag (ThermoSientific, MA1-91878-1MG, clone FG4R), RNAPII-CTD (sc-56767, clone 8WG16), RNAPII CTD-Ser2P (Bethyl, A300-654A), RNAPII CTD-Ser5P (Active Motif, 39749), RNAPII CTD-Ser7P (Millipore, 04–1570), NF-κB/p65 (sc-372), NF-κB2 (ProteinTech, 10409-2-AP), H2B (Millipore, 07–371), H3 (Millipore, 05–928), TBP (sc-273), TAF4 (sc-136093), Cyclin T1 (sc-10750), CDK9 (sc-8338), RNF20 (ProteinTech, 21625-1-AP), KDM4A (Novus, NB110-40585), KDM4B (Novus, NB100-74605), KDM4C (NBP1-49600), KDM4D (NBP1-03357), KDM5A (NB110-40499), KDM5B (NB100-97821), JMJD6 (NBP1-71693), NELF-A (sc-32911), Csn3 (sc-100693), Csn8 (sc-393482), PRMT6 (ProteinTech, 15395-1-AP), SHMT1 (ProteinTech, 14149-1-AP), SHMT2 (ProteinTech, 11099-1-AP), BRCC36 (Novus, NBP1-76831), Ubquitin (Millipore, 05–944), K48Ub (Millipore, 05–1307), K63Ub (Millipore, 05–1313), p62 (ProteinTech, 18420-1-AP), Rabbit IgG (sc-2027), Mouse IgG (sc-2025) and HRP-conjugated 2nd antibodies (sc-2004, sc-2005 & sc-2033).

### JIB-04 target identification using DiffPOP (Differential Precipitation of Proteins)

Five 15-cm plates of 2D10 T cells (Jurkat T cells, clone 2D10, as described in [5, Table 1], Dr. Jonathan Karn lab, Case Western Reserve University) were collected and washed by PBS. Cells were lysed in Phosphoprotein Kit Buffer A (Clontech 635626). An aliquot of 0.5 ml of whole cell lysate was centrifuged at 18000 x g for 10 min on ice, and the supernatant was collected as the initial lysate. To this lysate we added 2 μl of DMSO or JIB-04 (50 mM in DMSO), respectively. Proteins were differentially precipitated in ten fractions by the stepwise addition of 90% methanol 1% acetic acid. Protein pellets were then washed in cold acetone, solubilized and digested in a standard tryptic digest. Digested samples were analyzed by mass spectrometry. The differential distribution of cellular factors precipitated from extracts treated with DMSO or JIB-04 were calculated using the Pearson’s correlation. The most negative values of the Pearson’s correlation represent those proteins whose solubility is most altered by exposure to JIB-04, which implies a direct drug-protein interaction. For verification, protein fractions were analyzed directly by immunoblot using an SHMT2-specific antibody.

### RNA extraction, qRT-PCR, RNA-seq and bioinformatic analysis

Total RNA was extracted using RNeasy Plus Mini Kit (Qiagen). RNA was then quantitated by NanoDrop. About 1 μg of total RNA was subjected to reverse transcription using SuperScript III First-Strand Synthesis System kit from Invitrogen. About 0.5 to 1 μl of final cDNA products were used as PCR templates in each well of 96-well plate (ABI). The CTs from the real time PCR using ABI 7300 system were analyzed by Excel (The PCR efficiency was set to 2). For RNA-seq, purified RNA samples were first treated with Dnase I and purified by Zymo RNA purification kit (Zymo Research). HiSeq SE50 was performed on samples by the Next Generation Sequencing Core Facility at Salk Institute. The raw RNA-seq data can be accessed through NCBI GEO database (Accession: GSE109460). Sequencing reads were aligned to the human hg19 and HIV genomes using STAR and human gene-level read counts were obtained with feature Counts. Genes with less than 10 reads in all samples were ignored in further analysis. The reads were separately mapped to HIV-1 and d2EGFP sequences with BWA. From the uniquely-aligned read counts, the differential gene expression was calculated using DESeq2 and a design formula that accounted for sample batch, TNFα treatment, and JIB-04 treatment. For differentially expressed genes with at least a 2-fold change with adjusted p-value of 0.05 or less, Gene Ontology enrichment for biological process terms was carried out using clusterProfiler. The ten most significantly enriched terms per sample were included, but only terms with 200 associated genes or fewer were shown. For heatmap, all raw read counts were first transformed using a regularized logarithm (rlog) to generate log2-scaled values. The 100 genes with the largest differences between minimum and maximum rlog values were plotted after mean-centering. (See S1 Supporting Methods_PDF for other Methods).

## Supporting information

S1 Fig The lentiviral vector, JIB-04 effect on cell numbers, experimental procedures and chemical structure of JIB-04 (Related to Fig 1). **(A)** Schematic diagram of the HIV-1 lentiviral vector integrated into the MSRB1 site of 2D10 Jurkat T cells. **(B)** Total protein concentrations of whole cell extracts from 2D10 or HeLa cells (P4 and Tet-on-Tat-off) treated with different concentrations of JIB-04 after 24 h. **(C)** Schematic experimental procedures used in Fig 1. **(D)** Chemical structure of JIB-04.
(TIF)

S2 Fig Schematic experimental protocol of ChIP (Related to Fig 2). ChIP protocol: Cells were pre-treated with DMSO or 3 μM of JIB-04 for 16 h. Cells were then stimulated by TNFα (10 ng/ml) for 0 h (blue line), 0.5 h (pink line), 2 h (yellow line), and 6 h (light blue line) before ChIP, respectively. ChIP antibodies were described in Methods.
(TIF)

S3 Fig JIB-04 has only minor effects on host cell transcription at low concentration (Related to Fig 3). **(A)** Pie-chart of the 811 genes (out of 13546 genes with reads >10) altered more than 2-fold by JIB-04 (p<0.05). These 811 genes were listed in S1 File. **(B)** The top ten Gene Ontology enrichment biological process terms for DMSO vs JIB-04 and TNFα 0 h vs 6 h. **(C)** qRT-PCR results for the indicated genes randomly-selected from the top 100 heatmap for histones and JIB-04 activated genes, respectively. The significant differences between DMSO-treated and JIB-04-treated samples were analyzed by Student’s T-test (*** = p<0.0005). **(D)** Immunoblot analysis of histone H2B and H3 protein levels in 2D10 cells that were exposed to JIB-04 (0–10 μM) for 24 h. Csn3 served as loading control.
(TIF)

S4 Fig JIB-04 inhibited HIV replication with high cell toxicity in primary CD4+ T cells (Related to Fig 4). Graph show the data of analyzing JIB-04 in primary CD4+ T cells. The percentage of intracellular HIV-p24 was used to monitor the inhibition effect of the compounds. No treatment with HIV infection sample was set as negative control. DMSO plus 500 nM of commercial HIV-drug Raltegravir-treatment sample was set as positive control. The inhibition% values of the Y-axis were calculated by the formula (inhibition% = (p24% of no treatment–p24% of the respective treatments) / p24% of no treatment*100%). Raltegravir treatment reached 100% inhibition so as high concentrations of JIB-04. The negative value of DMSO-treatment showed DMSO treatment promoted infection. The viability of primary T cells was shown by the orange line.
(TIF)

S5 Fig Knockdown of various KDMs failed to recapitulate the effect of JIB-04 on Tat expression (Related to Fig 5). **(A)** One representative immunoblot for the indicated factors at the conditions of knocking down the JMJDs/KDMs in 2D10 cells (KDM4D, Csn3, USP7, Csn8 and Cyclin T1 as loading controls). **(B)** One representative immunoblot for the indicated factors at the conditions of knocking down KDM4C in Tet-on-Tat-off HeLa cells (Cyclin T1, loading control). **(C)** Schematic diagram of protocol for panel A in 2D10 cells.
(TIF)

S6 Fig JIB-04 increases proteolytic destruction of Tat protein (Related to Fig 6). **(A)** Titration of JIB-04 in Tet-on-Tat-off HeLa cells. Top, immunoblot for the inidcated proteins at the concentrations of JIB-04. Cyclin T1 served as loading control. Bottom, qRT-PCR for HA-Tat86 mRNA levels at the same concentrations of JIB-04 as in top penal. Tat mRNA was normalized to *ACTB* mRNA and Tat mRNA treated with Doxycycline was normalized to 1. **(B)** Top, Dual-Luc assay analysis for HIV-LTR-Luc at the indicated treatments in Tet-on-Tat-off HeLa cells. HIV-LTR-Luc was normalized to SV40-Renilla-Luc. Middle, Luc assay analysis for SV40-Renilla-Luc at the same condition. Renilla-Luc activity was normalized to total protein concentrations. Bottom, CMV-β-Gal assay for CMV-β-Gal at the same condition. CMV-β-Gal activity was normalized to Renilla-Luc activity. Activity from cells treated by 10 μg/ml doxycycline was normalized to 1. The significant differences between luciferase and β-Gal activity for DMSO and JIB-04 treated samples were calculated by Student’s T-test (ns = non-significant, *p<0.05). **(C)** Left, immunoblot results showed the half life of the indicated proteins in 2D10 T cells treated by 1 μM cycloheximide (Chx) and pre-treated with DMSO or 5 μM JIB-04 for 1 h. Cyclin T1 served as loading control. Right, relative levels of Tat was measured by Image J and graphed.
(TIF)

S7 Fig Hydroxychloroquine prevents Tat protein degradation in Tet-on-Tat-off HeLa cells (Related to Fig 7). **(A)** Immunoblot analysis of the indicated factors in the presence of increasing concentrations of Hydroxychloroquine in Tet-on-Tat-off HeLa cells. Cyclin T1 served as loading control. **(B)** Immunoblot analysis of HIV-1 Tat in 2D10 cells exposed to another autophagy inhibitor, 3-Methyladenine (3-MA). Cyclin T1 served as loading control.
(TIF)

S8 Fig Knockdown of BRISC and SHMT1/2 decrease Tat protein but not mRNA levels and each of single lysine or arginine mutations on Tat is not sufficient to prevent its destruction by JIB-04 (Related to Fig 8). **(A)** Top, schematic diagram of the protocol used. Bottom, immunoblot for the indicated factors under the indicated treatments (Cyclin T1 and TAF4 as loading controls). **(B)** qRT-PCR for mRNAs of indicated genes under the same treatments in penal A in 2D10 cells. The significant differences were analyzed by Student’s T-test (* = p<0.05, ** = p<0.005, *** = p<0.0005, ns = non-significant). **(C)** qRT-PCR to check the indicated mRNAs of the indicated genes after knocking down SHMT1 or BRCC36 in Tet-on-Tat-off HeLa cells. **(D)** Left, protein half-life of Tat was tested by treating with 1 μM cycloheximide for different durations by immunoblot (Cyclin T1 and Csn3 as loading controls). Right, Tat levels at the conditions of knocking down SHMT2 in 2D10 cells were calculated by Image J and averaged from three independent experiments. The significant differences between siCtrl and siSHMT2 were calculated by Student’s t-test (*p<0.05, **p<0.005). **(E)** Top, dual-Luc analysis of the activity of wild-type and mutant Tat in stimulating HIV-1 LTR promoter. Bottom, the expression levels of Tat WT and single lysine or arginine mutants were shown by immunoblot. Cyclin T1 served as loading control. **(F)** The effect of JIB-04 on wild-type or single lysine or arginine mutant Tat proteins was shown by immunoblot. Heat shock protein 60 (HSP60), TAF4 and cyclin T1 served as loading controls. **(G)** The expression levels of STREP-Tat WT, ∆K (+K41) and ∆K in HeLa cells from same amount of plasmids were shown by immunoblot. Cyclin T1 served as loading control.
(TIF)

S1 Supporting Methods Supplementary methods used in this study. (PDF)

S1 File RNA-Seq analysis of JIB-04. (XLS)

S2 File DiffPOP analysis of JIB-04 protein targets. (XLS)
