# Supplementary figures and images for: SHMT2 and the BRCC36/BRISC deubiquitinase regulate HIV-1 Tat K63-ubiquitylation and destruction by autophagy
Source: PLoS Pathog. 2018 May 23;14(5):e1007071. doi: 10.1371/journal.ppat.1007071 (PMC5988312; doi:10.1371/journal.ppat.1007071)

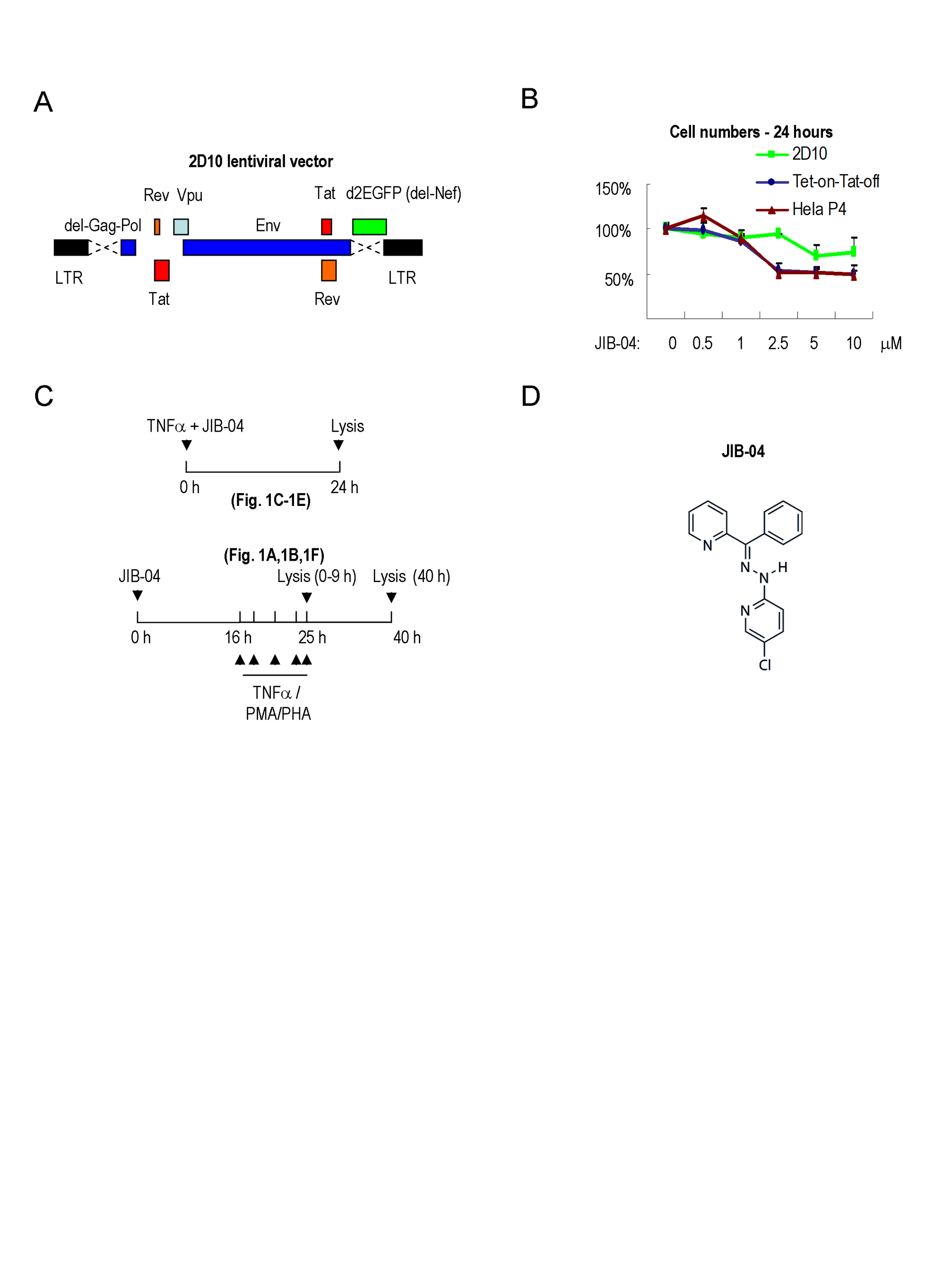

Supplement: S1 Fig — (A) Schematic diagram of the HIV-1 lentiviral vector integrated into the MSRB1 site of 2D10 Jurkat T cells. (B) Total protein concentrations of whole cell extracts from 2D10 or HeLa cells (P4 and Tet-on-Tat-off) treated with different concentrations of JIB-04 after 24 h. (C) Schematic experimental procedures used in Fig 1. (D) Chemical structure of JIB-04. (TIF) [file ppat.1007071.s001.tif]

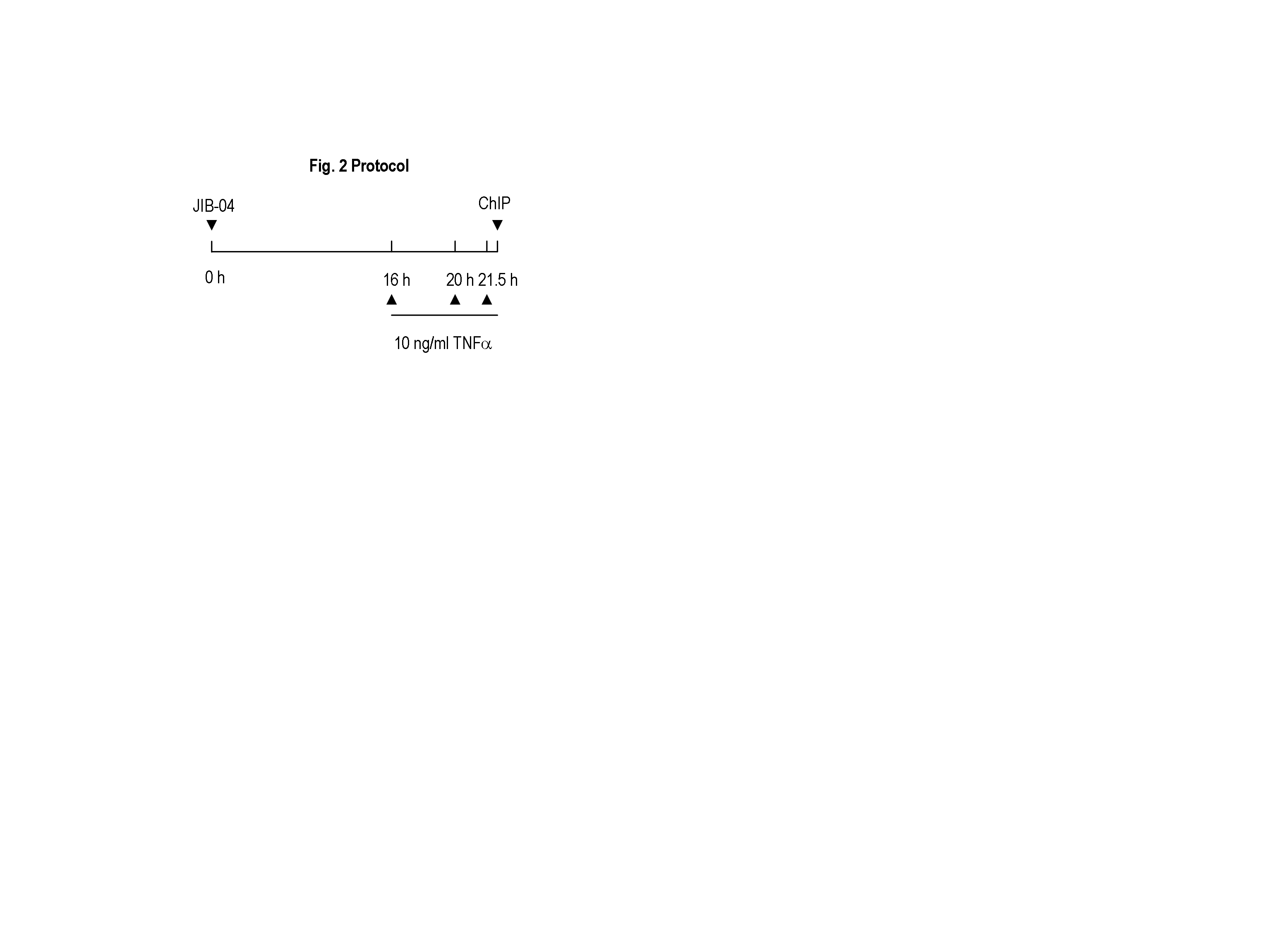

Supplement: S2 Fig — ChIP protocol: Cells were pre-treated with DMSO or 3 μM of JIB-04 for 16 h. Cells were then stimulated by TNFα (10 ng/ml) for 0 h (blue line), 0.5 h (pink line), 2 h (yellow line), and 6 h (light blue line) before ChIP, respectively. ChIP antibodies were described in Methods. (TIF) [file ppat.1007071.s002.tif]

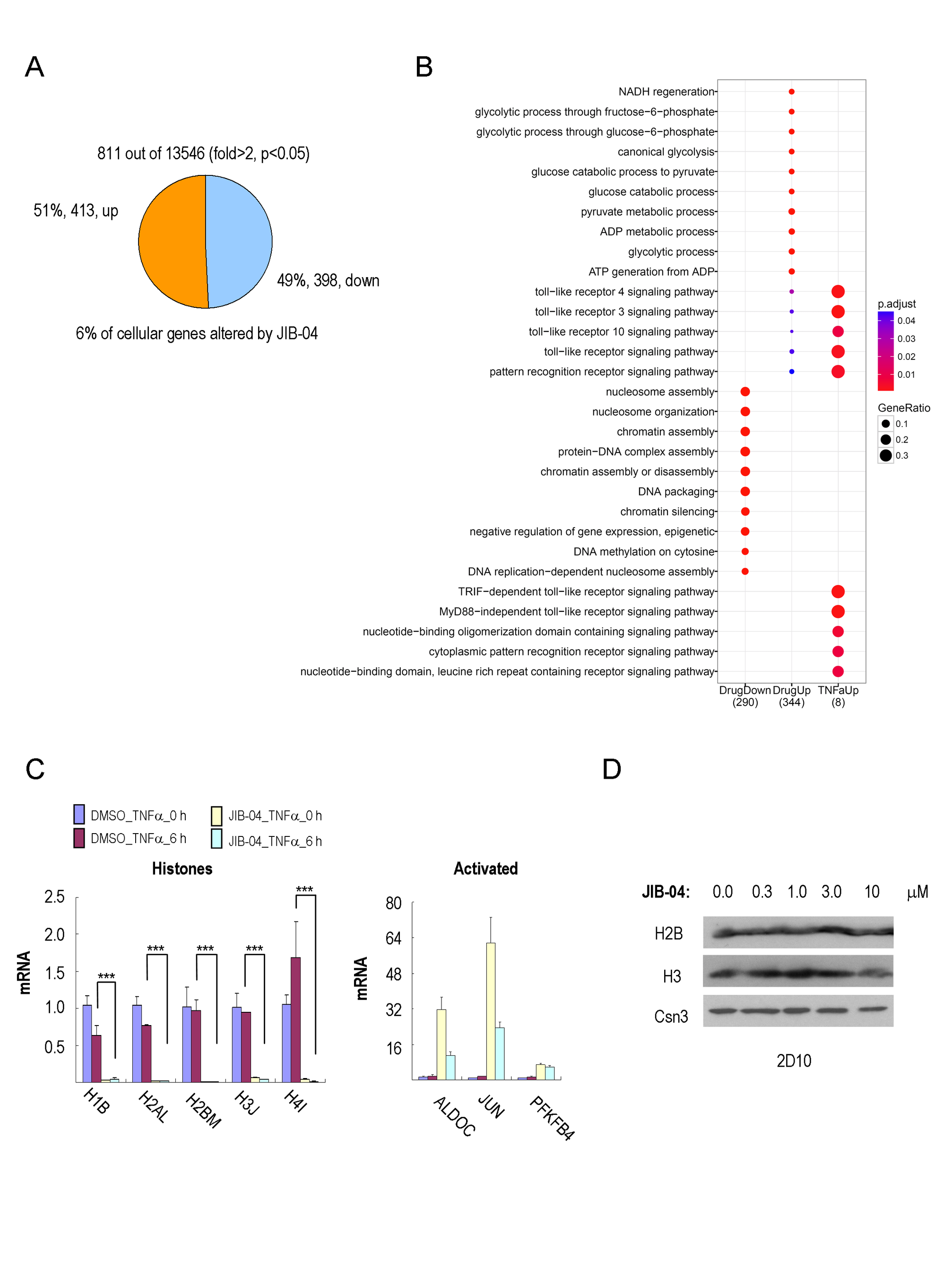

Supplement: S3 Fig — (A) Pie-chart of the 811 genes (out of 13546 genes with reads >10) altered more than 2-fold by JIB-04 (p<0.05). These 811 genes were listed in S1 File. (B) The top ten Gene Ontology enrichment biological process terms for DMSO vs JIB-04 and TNFα 0 h vs 6 h. (C) qRT-PCR results for the indicated genes randomly-selected from the top 100 heatmap for histones and JIB-04 activated genes, respectively. The significant differences between DMSO-treated and JIB-04-treated samples were analyzed by Student’s T-test (*** = p<0.0005). (D) Immunoblot analysis of histone H2B and H3 protein levels in 2D10 cells that were exposed to JIB-04 (0–10 μM) for 24 h. Csn3 served as loading control. (TIF) [file ppat.1007071.s003.tif]

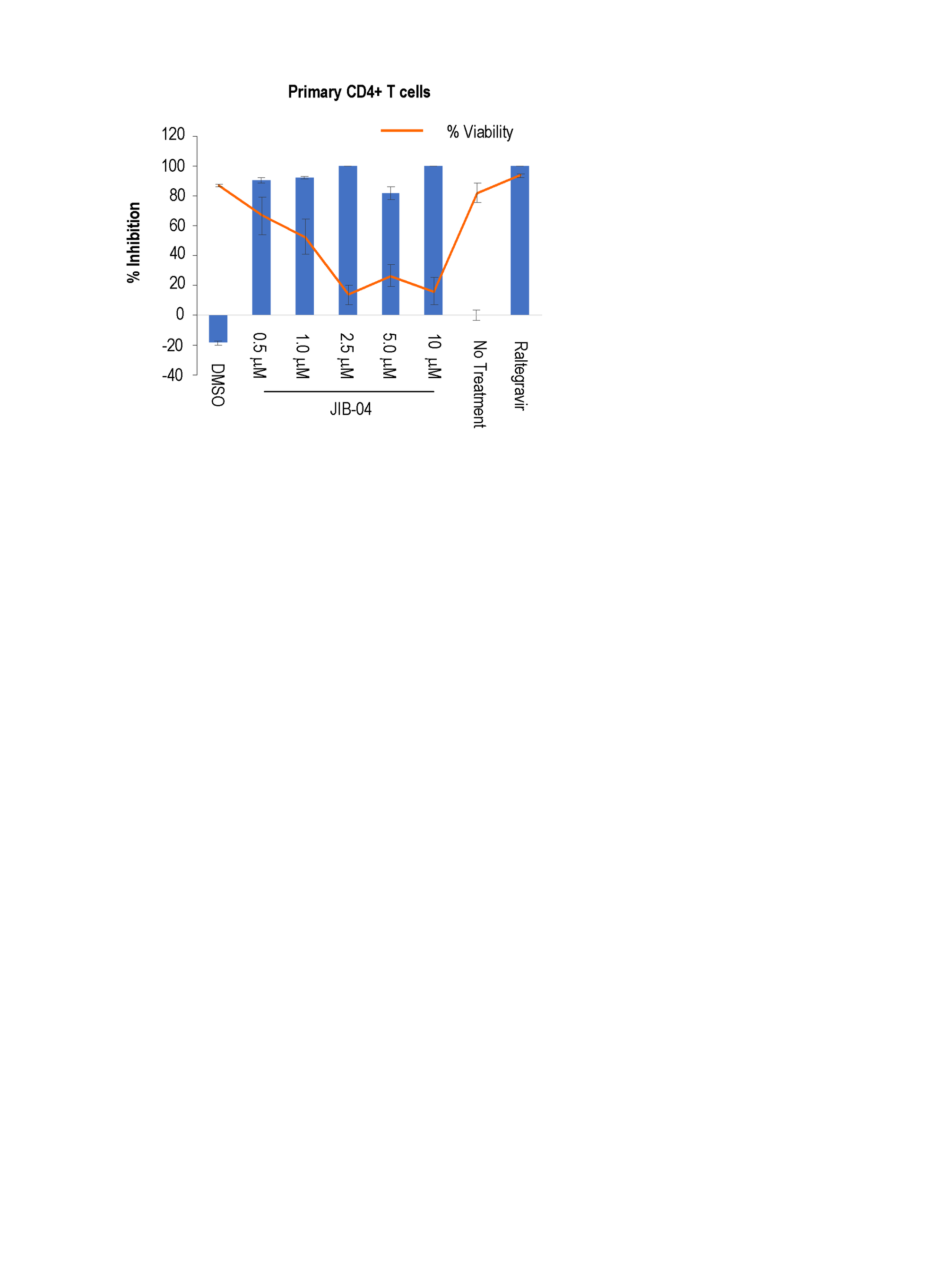

Supplement: S4 Fig — Graph show the data of analyzing JIB-04 in primary CD4+ T cells. The percentage of intracellular HIV-p24 was used to monitor the inhibition effect of the compounds. No treatment with HIV infection sample was set as negative control. DMSO plus 500 nM of commercial HIV-drug Raltegravir-treatment sample was set as positive control. The inhibition% values of the Y-axis were calculated by the formula (inhibition% = (p24% of no treatment–p24% of the respective treatments) / p24% of no treatment*100%). Raltegravir treatment reached 100% inhibition so as high concentrations of JIB-04. The negative value of DMSO-treatment showed DMSO treatment promoted infection. The viability of primary T cells was shown by the orange line. (TIF) [file ppat.1007071.s004.tif]

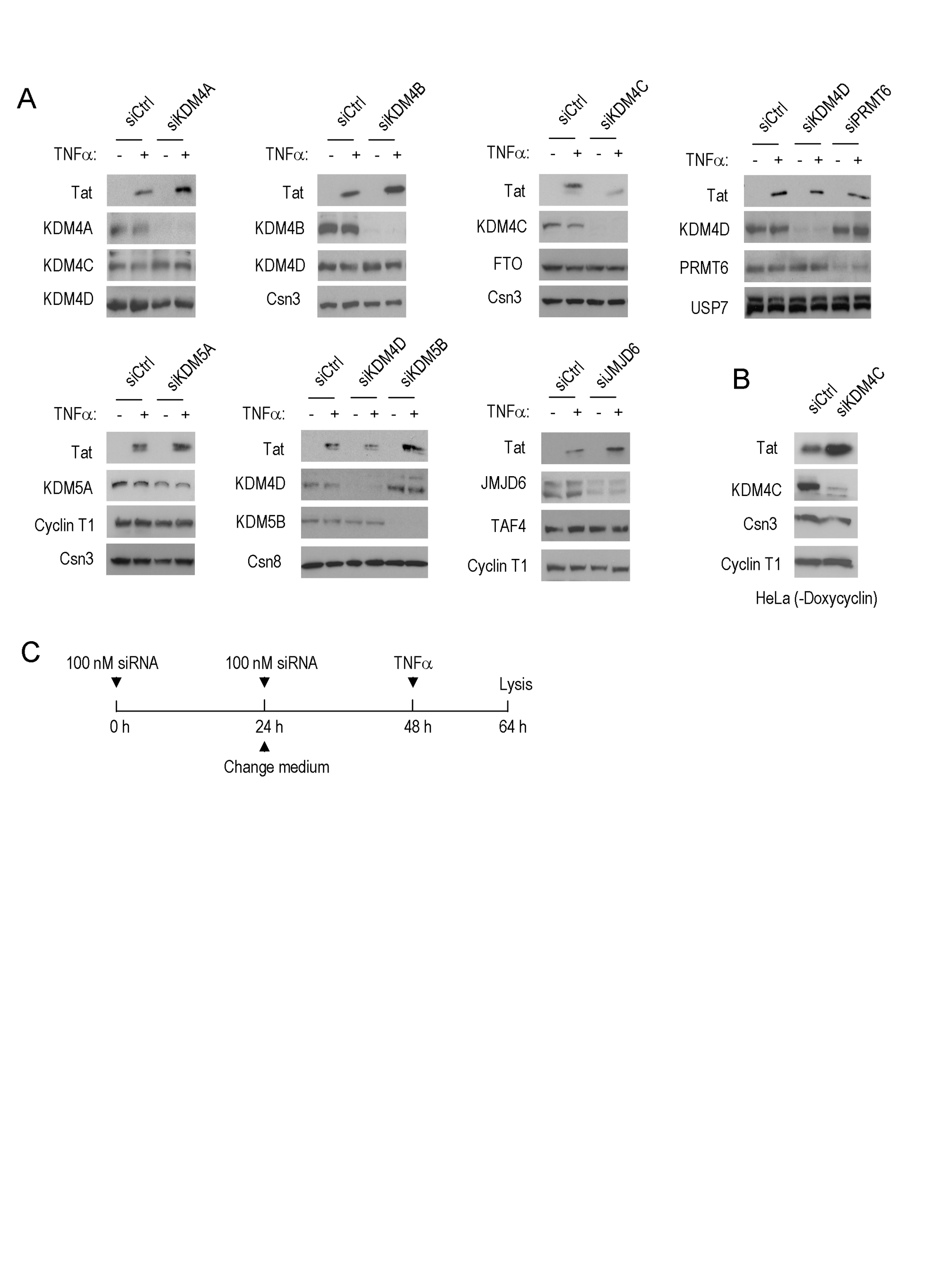

Supplement: S5 Fig — (A) One representative immunoblot for the indicated factors at the conditions of knocking down the JMJDs/KDMs in 2D10 cells (KDM4D, Csn3, USP7, Csn8 and Cyclin T1 as loading controls). (B) One representative immunoblot for the indicated factors at the conditions of knocking down KDM4C in Tet-on-Tat-off HeLa cells (Cyclin T1, loading control). (C) Schematic diagram of protocol for panel A in 2D10 cells. (TIF) [file ppat.1007071.s005.tif]

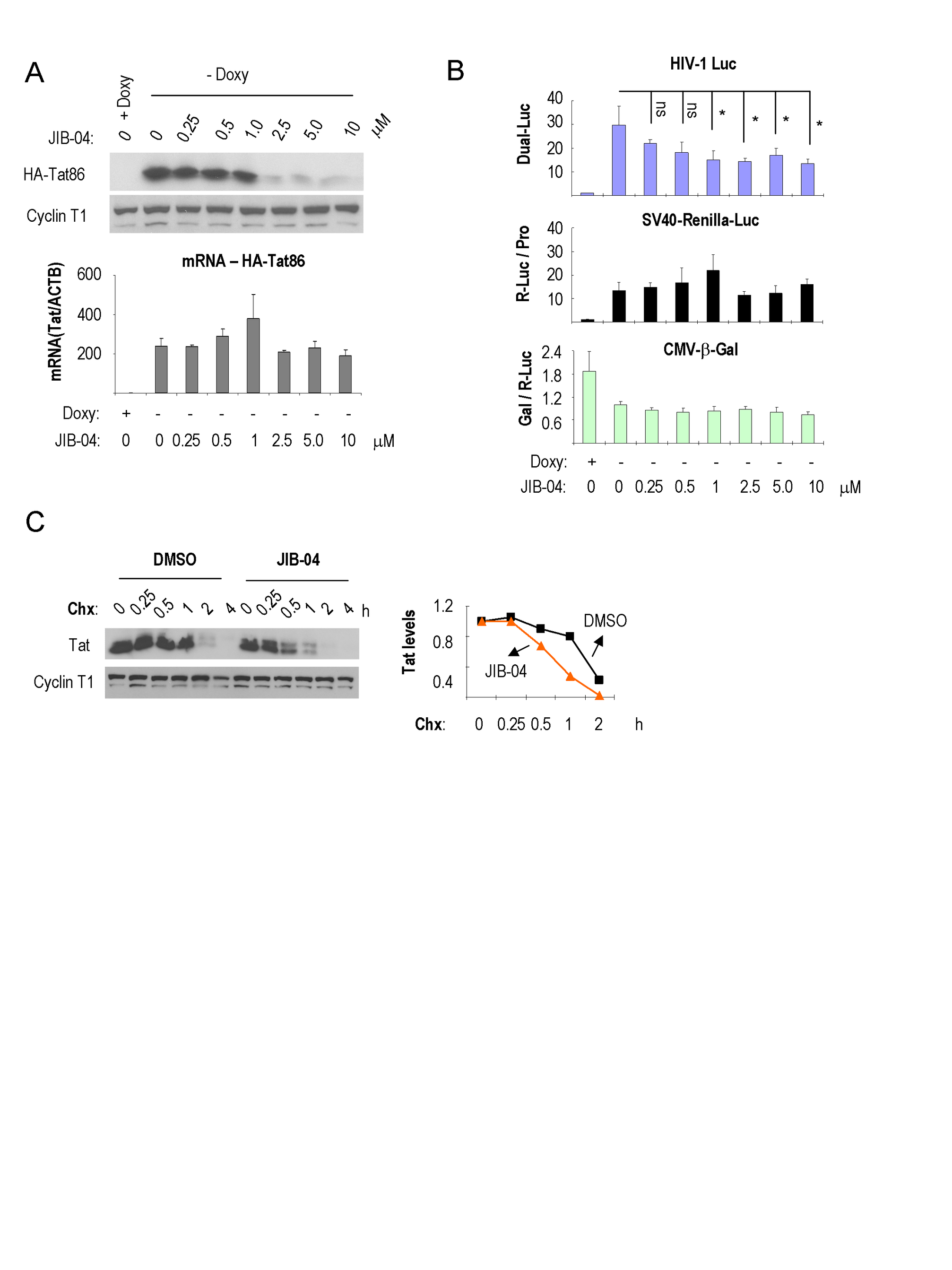

Supplement: S6 Fig — (A) Titration of JIB-04 in Tet-on-Tat-off HeLa cells. Top, immunoblot for the inidcated proteins at the concentrations of JIB-04. Cyclin T1 served as loading control. Bottom, qRT-PCR for HA-Tat86 mRNA levels at the same concentrations of JIB-04 as in top penal. Tat mRNA was normalized to ACTB mRNA and Tat mRNA treated with Doxycycline was normalized to 1. (B) Top, Dual-Luc assay analysis for HIV-LTR-Luc at the indicated treatments in Tet-on-Tat-off HeLa cells. HIV-LTR-Luc was normalized to SV40-Renilla-Luc. Middle, Luc assay analysis for SV40-Renilla-Luc at the same condition. Renilla-Luc activity was normalized to total protein concentrations. Bottom, CMV-β-Gal assay for CMV-β-Gal at the same condition. CMV-β-Gal activity was normalized to Renilla-Luc activity. Activity from cells treated by 10 μg/ml doxycycline was normalized to 1. The significant differences between luciferase and β-Gal activity for DMSO and JIB-04 treated samples were calculated by Student’s T-test (ns = non-significant, *p<0.05). (C) Left, immunoblot results showed the half life of the indicated proteins in 2D10 T cells treated by 1 μM cycloheximide (Chx) and pre-treated with DMSO or 5 μM JIB-04 for 1 h. Cyclin T1 served as loading control. Right, relative levels of Tat was measured by Image J and graphed. (TIF) [file ppat.1007071.s006.tif]

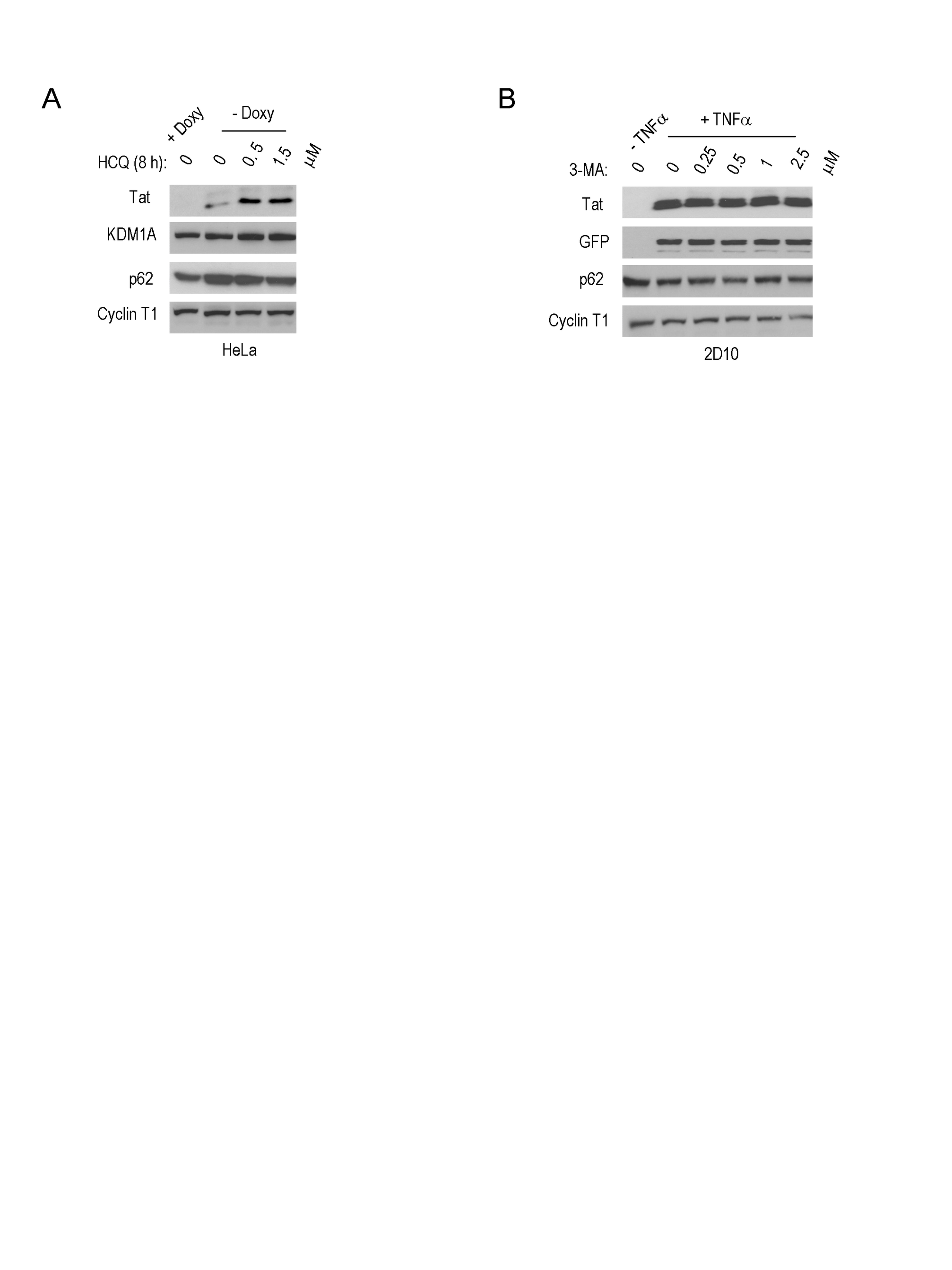

Supplement: S7 Fig — (A) Immunoblot analysis of the indicated factors in the presence of increasing concentrations of Hydroxychloroquine in Tet-on-Tat-off HeLa cells. Cyclin T1 served as loading control. (B) Immunoblot analysis of HIV-1 Tat in 2D10 cells exposed to another autophagy inhibitor, 3-Methyladenine (3-MA). Cyclin T1 served as loading control. (TIF) [file ppat.1007071.s007.tif]

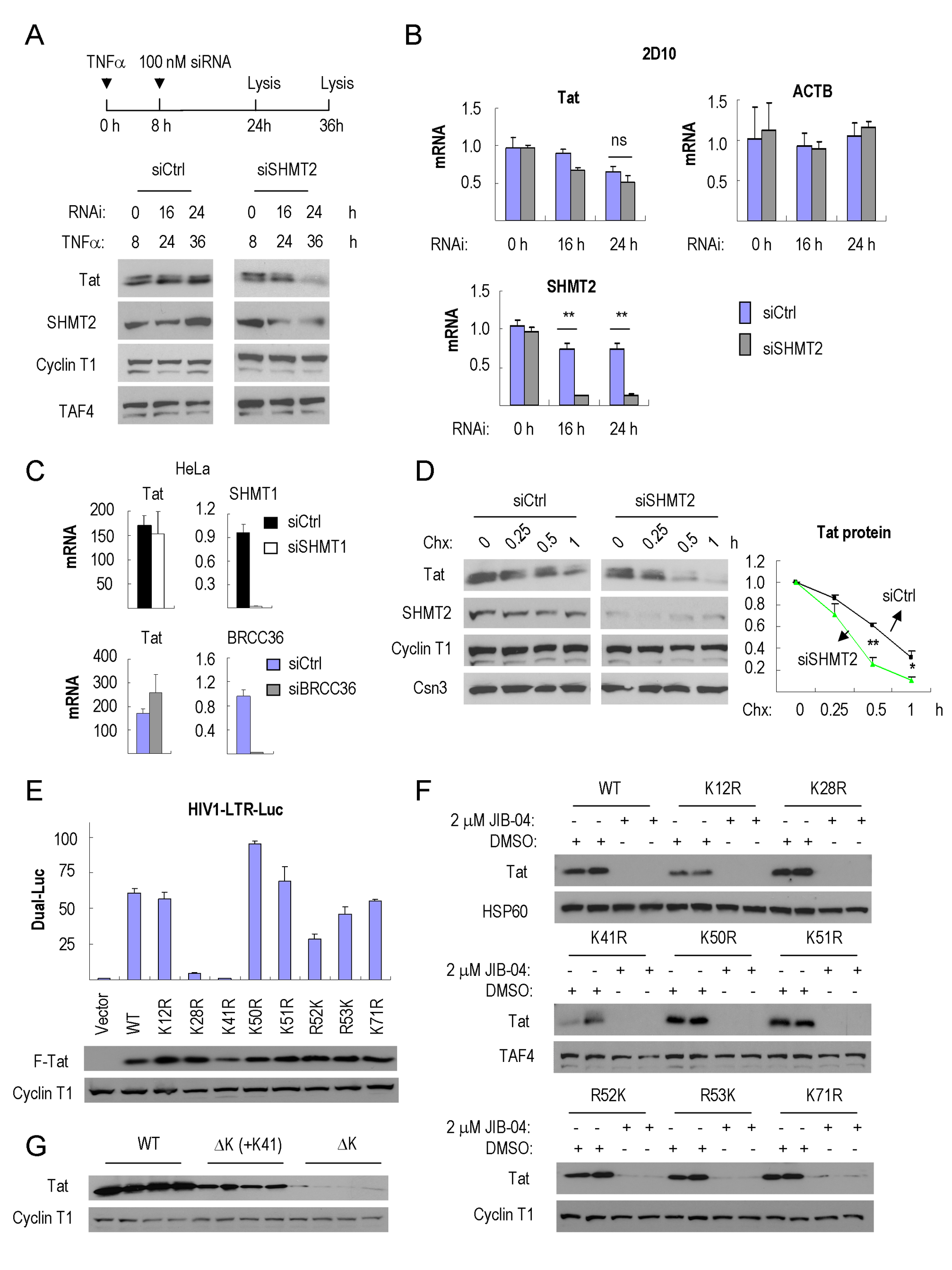

Supplement: S8 Fig — (A) Top, schematic diagram of the protocol used. Bottom, immunoblot for the indicated factors under the indicated treatments (Cyclin T1 and TAF4 as loading controls). (B) qRT-PCR for mRNAs of indicated genes under the same treatments in penal A in 2D10 cells. The significant differences were analyzed by Student’s T-test (* = p<0.05, ** = p<0.005, *** = p<0.0005, ns = non-significant). (C) qRT-PCR to check the indicated mRNAs of the indicated genes after knocking down SHMT1 or BRCC36 in Tet-on-Tat-off HeLa cells. (D) Left, protein half-life of Tat was tested by treating with 1 μM cycloheximide for different durations by immunoblot (Cyclin T1 and Csn3 as loading controls). Right, Tat levels at the conditions of knocking down SHMT2 in 2D10 cells were calculated by Image J and averaged from three independent experiments. The significant differences between siCtrl and siSHMT2 were calculated by Student’s t-test (*p<0.05, **p<0.005). (E) Top, dual-Luc analysis of the activity of wild-type and mutant Tat in stimulating HIV-1 LTR promoter. Bottom, the expression levels of Tat WT and single lysine or arginine mutants were shown by immunoblot. Cyclin T1 served as loading control. (F) The effect of JIB-04 on wild-type or single lysine or arginine mutant Tat proteins was shown by immunoblot. Heat shock protein 60 (HSP60), TAF4 and cyclin T1 served as loading controls. (G) The expression levels of STREP-Tat WT, ∆K (+K41) and ∆K in HeLa cells from same amount of plasmids were shown by immunoblot. Cyclin T1 served as loading control. (TIF) [file ppat.1007071.s008.tif]
