# Supplementary material for: SHMT2 and the BRCC36/BRISC deubiquitinase regulate HIV-1 Tat K63-ubiquitylation and destruction by autophagy
Source: PLoS Pathog. 2018 May 23;14(5):e1007071. doi: 10.1371/journal.ppat.1007071 (PMC5988312; doi:10.1371/journal.ppat.1007071)
Supplement: S1 Supporting Methods — (PDF) [file ppat.1007071.s009.pdf]

Supplementary Methods:

Plasmids

Plasmids HA-Ubiquitin WT (#17608), HA-Ubiquitin K63only (#17606), HA-Ubiquitin K48only (#17606) and Flag-HA-BRCC36 (#327340) were purchased from Addgene. Plasmid Flag-SHMT1 was cloned by inserting PCR fragments from plasmid pLJM5-SHMT1 (Addgene, #83904) into Hind III and Not I sites of pCDNA5 vector. The cloning primers: 5'-ATCG*aagctt*AAGATGGCAGACAGGGATGCCACC-3' for HindIII and 5'-ATCG*gcggccgc*TCAGAAGTCAGGAAGGCCAGG-3' for Not I. All of Flag-Tat single lysine mutant plasmids are generated by Quick-change PCR mutagenesis kit (Agilent 210515) using Flag-Tat101 WT plasmid as templates (Jones Lab) and confirmed by sequencing. The pimers for Quick-change PCR were listed in the table below. Plasmids STREP-Tat WT, STREP-Tat ΔK (no lysine) and STREP-Tat ΔK (+K41) were generously provided by Dr. Frankel Alan's lab at UCSF.

Cell culture, Induction of HIV-1, RNAi and Dual-luciferase assay

2D10 Jurkat T cells (Jurkat T cell, clone 2D10) were generously provided by Dr. Jonathan Karn (Case Western Reserve University). 2D10 cells were cultured in suspension by RPMI-1640 medium (Corning, 10-040-CM) plus 10% FBS (Omega Scientific Inc, FB-01) and 1% antibiotic-antimycotic solution (Corning, 30-004-CI) inside 37 ℃ incubator supplied with 5% CO_2. To knockdown factors by siRNA using 2D10 cells, about $0.5*10^6$ cells were first transferred to each well of 12-well plate. Cells were then transfected with 100 nM siRNA using Stemfect RNA transfection kit (Stemgent, 00-0069). Twenty-four hours later, medium was changed and cells were transfected again with 100 nM siRNA. Fourty-eight hours after the first siRNA transfection, rTNFα (R&D system, 210-TA-020, 10 ng/ml) was added to medium to induce HIV-1 expression for 8-16 hours. Cells were then collected, washed with PBS, lysed by 150 μL (per well) E1A lysis buffer (50 mM Hepes pH 7.9, 50 mM NaCl, 0.1% NP-40, 0.2 mM EDTA pH 8.0, 0.2 mM PMSF, 1-5 mM beta-mercaptoethanol, completed with H_2O) for 10 minutes on ice and analyzed by Western blot.

Tet-on-Tat-off HeLa cells (Jones lab) integrated with HA-Tat86 and HeLa P4 cells (Jones lab) integrated with HIV-1-LTR-Luc were cultured by DMEM medium (Corning, 10-017-CM) plus 10% FBS (Omega Scientific Inc, FB-01) and 1% antibiotic-antimycotic solution (Corning, 30-004-CI) inside 37 ℃ incubator supplied with 5% CO2. To carry out dual-luciferase assay, about $0.1*10^6$ cells were transferred to each well of 12-well plate and cultured for 6-16 hours to reach 70% confluency. At this point, cells were ready for transfection and inhibitor treatments. For Tet-on-Tat-off HeLa cells, cells were treated by DMSO or JIB-04 for one hour, followed by transfection of 100 ng HIV-1-LTR-Luciferase, 20 ng SV40-Renilla-Luciferase and 20 ng CMV-β-Galactosidase plasmids per well by 3 μL Lipofectamine2000 (Life Technologies, 11668019). For dual-luc assay using HeLa P4 cells, 100 ng of Flag-Tat-101 WT or mutants were co-transfected with 20 ng SV40-Renilla-Luciferase per well by 3 μL Lipofectamine2000. Dual-Luc assay was carried out 24 hours after transfection. To knockdown factors by siRNA in HeLa cells, about $0.1*10^6$ cells were transferred to each well of 12-well plate and cultured for 6-16 hours to reach 50% confluency. At this point, cells are ready for transfection. Final concentration of 50-100 nM siRNA together with or without different amounts of plasmids as indicated were transfected only once into HeLa cells using Lipofectamine2000. Fourty-eight hours after transfection, cells were lysed by 150 μL E1A lysis buffer or 1X Passive Lysis Buffer (Promega, E1941) for 10 minutes on ice. Lysates were then spined at 4 ℃ for 10 mins and the supernatant was ready for Dual-Luc assay (Promega, E1960) using Luminoskan™ Ascent Microplate Luminometer. Dual-Luc assay was carried out by adding 20 μL lysate to a 96-well plate first, followed by adding 50 μL firefly luciferase substrate and 50 μL Renilla luciferase substrate to measure luciferase activity, respectively. Firefly luciferase activity was normalized to Renilla luciferase activity. CMV-beta-Galactosidase assay was previously described (Brasier et al., 1989) and Galactosidase activity was normalized to Renillla luciferase activity. Partial cells were analyzed by western blot to determine the protein level.

Inhibitor treatments

All inhibitors were purchased from Selleckchem except doxycycline and cycloheximide (Sigma, D3447 and C7698, respectively). The catalogs for the inhibitors are JIB-04 (S7281), ML324 (S7296), IOX1 (S7234), GSK J1 (S7581), 2-PCPA (S4246), MG132 (S2619), Flavopiridol (S2679), Hydroxychloroquine (S4430) and 3-Methyladenine (3-MA, S2767), respectively. All inhibitors were diluted in DMSO or H2O according to manufacturer's instructions. To determine the IC50 of the inhibitors, 2D10 cells were treated with 0-50 μM final concentration of indicated inhibitors and incubated for 24 hours in the presence/absence of 10 ng/ml TNFα (R&D, 210-TA). Twenty-four hours later, cells were collected and lysed by E1A lysis buffer and the supernatant after spinning was used for immunoblot. To test the effect of JIB-04 on HIV-1 expression, DMSO or 3-μM JIB-04 was added to cells for over night (16 h). Next morning, TNFα (10 ng/ml) or PHA/PMA (10 μg/ml and 50 ng/ml, respectively, Sigma, L8754-5mg/P1585-1MG) was added to 2D10 cells at different time points. Cells were collected for immunoblot or for RNA extraction (Qiagen RNeasy plus kit, 74134). To test the effect of JIB-04 on HIV-1 Tat protein degradation at early time points in 2D10 cells, TNFα (10 ng/ml) was added to 2D10 cells to induce Tat expression over night (16 h). Next morning, cells were treated for short durations (e.g. 8 h) with 5-μM JIB-04, 5-μM MG132, 2.5-μM Hydroxychloroquine or their mixtures, respectively. To test the effect of JIB-04 on Tat protein degradation in Tet-on-Tat-off HeLa and HeLa-P4 cells, JIB-04, MG132 or both were added to HeLa cells at the time of plasmid transfection. Twenty-four hours later, cells were collected and analyzed by Dual-Luc assay, immunoblot and rt-PCR.

Co-Immunoprecipitation (Co-IP) and Chromatin Immunoprecipitation (ChIP)

The expression of HA-Tat was turned off by adding 10 μg/ml doxycycline to the medium of Tet-on-Tat-off HeLa cells. Twenty-four hours after incubation, cells were washed once by cold PBS and lysed by E1A lysis buffer (1 mL per 10-cm plate) for 10 minutes on ice. Cells were then scrapped and spinned at 13200 rpm for 10 mins. The supernatant from samples were adjusted to similar concentration by diluting with E1A

lysis buffer. For Co-IP, about 500 μL of E1A lysate (~1.5 mg total protein in 250 mM salt) was diluted by adding 210 μL of BC-0 (20 mM Hepes, pH 7.9 at 4 ℃, 20% glycerol, 0.2 mM EDTA, 0.1% NP-40, 0.2 mM PMSF and 5 mM β-Mercaptoethanol) to reach final salt concentration at 175 mM. Next, about 50 μL of HA-Agarose slurry (Pierce, 26181) was added to the lysate and the mixture was incubated in cold room over night. Next morning, lysate was spinned for 10 second at 3000 g and the unbound was disposed. Remaining HA-beads were transferred to a new tube and washed three times with BC-150 (20 mM Hepes, pH 7.9 at 4 ℃, 150 mM KCl, 20% glycerol, 0.2 mM EDTA pH 8.0, 0.1% NP40, 0.2 mM PMSF and 5 mM β-Mercaptoethanol). Beads were then heated at 95 ℃ for 5 minutes and analyzed by SDS-PAGE western blot.

Endogenous Tat-ubiquitination assay (Fig 6D) was performed by incubating 5 μg of anti-Tat antibodies (ab43014) with 1.5 mg cell lysate from Tet-on-Tat-off HeLa cells at 400 mM salt for at least seven hours, followed by adding 50 μl Protein G slurry (Pierce/20398) beads to incubate in the cold room over night. The following morning, beads were washed twice by BC-500 (500 mM KCl) and once by BC-150, respectively. SDS loading buffer were added and heated to elute Tat from beads. Half of elution was analyzed by SDS-PAGE and bloted with mouse monoclonal anti-Ubiquitin antibodies (Millipore 05-944 for Ubiquitin, 05-1307 for K48Ub or 05-1313 for K63Ub, respectively) to detect ubiquitin. The other half was bloted with mouse anti-Tat antibody (ab42359). For ectopic Tat-ubiquitination assay, HeLa P4 cells were transfected with 5 μg of Flag-Tat-101 (Jones Lab) together with 5 μg of HA-Ubiquitin WT (Addgene 17608), HA-Ubiquitin K63only (Addgene 17606) or HA-Ubiquitin K48only (Addgene 17606), respectively for 24 hours using Lipofectamine2000 in one 10-cm plate, followed by treating with 1 or 3 μM of JIB-04 for 12 hours (Fig 6E) or 100 nM siRNA for 24 hours (Fig 8E) before lysis. Lysates were IP at 175 mM salt together with anti-Flag resin (Sigma, M8823) over night in cold room. The following morning, resins were washed three times with BC-500 (500 mM) and once with BC150 (15 mins/each wash) before immunoblot.

The detailed ChIP protocol has been described previously (Chen et al., 2014). Briefly, formaldehyde (Sigma, F8875) was added to 10 ml of 2D10 cells (5-10*10^6) in a 10-cm plate to reach final concentration at 1%. Cells were rotated slowly at room temperature (RT) for 10 minutes, followed by adding 500 µl of 2.5 M glycine to stop crosslinking. Cells were then washed twice by ice-cold PBS. After washing, cells were resuspended in 400-600 µl ChIP lysis buffer (1% SDS, 10 mM EDTA, 50 mM Tris-HCl pH-8.1) and incubated on ice for 10 mins. Sonication was carried out four times (15 seconds/time, with 1 min interval) on ice at output-3 using Fisher Sci 550 Sonic Dismembrator. Lysate was centrifuged at 14,000 g for 10 min at 4 °C and the supernatant was collected in a new tube. At this point, 20 µl lysate was set aside as input. About 100-400 μl of lysate was diluted (1:10) by IP dilution buffer (Chen et al., 2014) followed by adding antibodies (0.5-5 μg) and incubated overnight in cold room. The following morning, 40-60 µl Protein G slurry beads (Pierce, 20398) were added to lysate and rotated inside cold room for more than three hours. Beads were then washed and eluted in 150 μl elution buffer (1% SDS, 0.1M NaHCO3). For reverse-crosslinking, 6 μl of 5 M NaCl was added to the elution and incubated at 65 °C over night. At last step, DNA (including inputs) was purified with quick Spin PCR Purification Kit (Qiagen, 28106) and eluted in 100 µl H2O for q-PCR amplification.

Identification of Tat–p62 interaction by MudPIT

The detailed method of MudPIT and the Tat-interacting protein list were previously published (Chen et al. 2014). The autophagy-related HA-Tat-interacting protein factors were selected for the list in Fig 7A.

Blue cell assay

The blue cell assay for HIV infectivity was previously described (Day et al., 2006). Briefly, Hela P4.R5 MAGI. cells containing the β-galactosidase indicator under the control of the HIV-1 LTR were plated at a density of 2×10^4 cells per well in a 48-well tissue culture plate. On the following day, cells were infected in triplicate with 100 μl of virus-containing supernatants from HEK293T cells. After a 2 h incubation, the

volume was increased to 500 μl by adding 400 μl of complete media with DMSO or JIB-04. Following a 2-day incubation at 37 ◦C, 5% CO2, the cells were fixed with 1% formaldehyde, 0.2% glutaraldehyde in PBS for 5 min at room temperature and stained in an X-gal (5-bromo-4-chloro-3-indolyl-β-d-galactopyranoside) solution overnight at 37 ◦C in a non-CO2 incubator. Finally, cells were washed once with PBS, once with water and allowed to air dry. Infectivity was assessed by counting the blue-stained, HIV-infected foci by eyes through a microscope (simple-count).

Test of JIB-04 in Primary CD4+ T cells

Peripheral blood from a healthy donor was collected by venipuncture according to institutional review board approved protocols into vacutainer tubes containing sodium heparin. Primary CD4+ T cells were isolated using RosetteSep CD4+ T-cell enrichment cocktail (StemCell Technologies Inc., Vancouver, Canada). Primary CD4+ T cells were incubated overnight at 37°C, 5% CO2 in RPMI 1640 medium supplemented with 5% human AB serum and were subsequently infected with wild-type NL4-3 virus. After 4 hours of infection, cells were resuspended to a concentration of 0.5 million cells per 1.5 ml media for treatment with JIB-04 drug at various concentrations. Cells treated with 500 nM Raltegravir was used as a control along with DMSO only, infected only and uninfected only controls. These cells were then plated on CD3/CD28 coated plates for proliferation and virus spread for 3 days after which they were de-plated and re-plated in a media containing IL-2 and IL-15 for another 4 days. Cells were harvested for intracellular HIV-P24 assay on Day 3 and Day 7 using ebioscience intracellular Fix/Perm kit.

The sequences of siRNAs (from Life Technologies):

siRNA	ID	Sense (5′- 3′)	Anti-Sense (5′- 3′)
Control	N/A	4390844, SilencerR-select Negative Control#1	
CCNT1	s2541	CGACCCAGACAAUAGACUAtt	UAGUCUAUUGUCUGGGUCGtg
NELF-A	s14859	CGCUUAACCUGGAGCUGGAtt	UCCAGCUCCAGGUUAAGCGag
PRMT6	s30337	GAGGCAAGACGGUACUGGAtt	UCCAGUACCGUCUUGCCUCgc
KDM4A	s18637	GCGACAAUCUUUAUCCUGAtt	UCAGGAUAAAGAUUGUCGCtg
KDM4B	s22867	GUCCCGAGCUGGUCAAUGAtt	UCAUUGACCAGCUCGGGACgg
KDM4C	s22990	GUAUUGAAGAAAUAUGGUAtt	UACCAUAUUUCUUCAAUACtg
KDM4D	s31267	AGAGAGACCUAUGAUAAUAtt	UAUUAUCAUAGGUCUCUCUgg
KDM5A	s11836	GCGAGUUUGUUGUGACAUUtt	AAUGUCACAACAAACUCGCca
KDM5B	s21145	GGCAGUAAAGGAAAUCGAAtt	UUCGAUUUCCUUUACUGCCgt
JMJD6	s23290	GGUGAACACCCUAAAAGAAtt	UUCUUUUAGGGUGUUCACCat
SQSTM1	13999	GGUGAAACACGGACACUUCtt	GAAGUGUCCGUGUUUCACCtt
SHMT1	14219	GGGUUGGAUUGGAGCUGAUtt	AUCAGCUCCAAUCCAACCCtc
SHMT2	S12823	CCACUACUCACAAGACUCUtt	AGAGUCUUGUGAGUAGUGGtg
BRCC36	241826	CAUGUUGAUGUUCGCACACtt	GUGUGCGAACAUCAACAUGtg

Primers for qRT-PCR, quick-change-PCR and ChIP:

**qRT-PCR primers**	**Sequences (5′-3′)**
Tat-F1	GAGCCAGTAGATCCTAGACTA
Tat-R1	GAGATGCCTAAGGCTTTTGTC
Tat-R2	CTTCGGGCCTGTCGGGTCCCC
Env-F	GCAGTGGGAATAGGAGCTTTGT
Env-R	GCTGCGCCCATAGTGCTT
Gag-F	AGCAACCCTCTATTGTGTGCAT
Gag-R	TGCGGTGGTCTTACTTTTGTTT
Rev-F	GACGATCTGCGGAGCCTGTGC
Rev-R	CTGCGTCCCAGAAGTTCCACA
d2GFP-F1	AGATCCGCCACAACATCGAG
d2GFP-R1	GTCCATGCCGAGAGTGATCC
d2GFP-F2	AAGGACGACGGCAACTACAA
d2GFP-R2	TTGTACTCCAGCTTGTGCCC
CXCL10-F	GCTTCCAAGGATGGACCACA
CXCL10-R	GCAGGGTCAGAACATCCACT
NFkB2-F	GAGGGCCTTTAGCGGACAG
NFkB2-R	CGGGTCCGCGTATCTTTGTA
NFkBIA-F	GAAGTGATCCGCCAGGTGAA
NFkBIA-R	CTGCTCACAGGCAAGGTGTA
IER3-F	CACCATGTGTCACTCTCGCA
IER3-R	AGAAGCCTTTTGGCTGGGTT
H1B-F	GTGGCGAAGAGCCCTAAGAA
H1B-R	TGGCCTTTGCAGCTTTAGGT

H2AL-F	AGAAGACCCGCATTATCCCG
H2AL-R	AGAGCCGTTGTATTGGAAAGTGA
H2BM-F	GACACCGGCATCTCTTCCAA
H2BM-R	TCACGGCGGAACTGTTACTG
H3J-F	CTGTGCTATTCACGCCAAGC
H3J-R	ACCCAAGATAGAGCAGGGGA
H4I-F	TCCAGGGTATCACCAAGCCA
H4I-R	ATGACTGGGCTTCAAAATGCC
CDK9-F	GGTGAGGGAATTGGTGAGGG
CDK9-R	CGTCCAAACTACTGCCACCT
TBP-F	GAGTTCCAGCGCAAGGGTTT
TBP-R	GGGTCAGTCCAGTGCCATAA
KDM4C-F	CTGCTGAGGGAGAAGTCGTC
KDM4C-R	GCATGTCAGAGGAAATCGAGC
ACTB-F	TGACGGGGTCACCCACACTGTGCCCA
ACTB-R	CTAGAAGCATTTGCGGTGGACGATGGAGGG
CCNK-F	CCATATACAAGATGGCCGCAGT
CCNK-R	GTGTGGTCCAGGTTTGCTGA
ALDOC-F	TCACGTAGCTCTGCGACATC
ALDOC-R	CACTTGGCAAAGTCAGCACC
JUN-F	GCTGCTCTGGGAAGTGAGTT
JUN-R	TTTCTCTAAGAGCGCACGCA
PFKFB4-F	CTCATTGTCATGGTGGGCCT
PFKFB4-R	TTTTAGCGGTGGATGGGCTT
SHMT1-F	AAGTTCGGGGTTTGGGGTTG
SHMT1-R	TGGTTCGAAGCTGCCTAGC
SHMT2-F	ATGTCTGACGTCAAGCGGAT
SHMT2-R	GGCCAGTTTTGGGGTTGAGC
BRCC36-F	CTGAAATGCGCACAGTTGCT
BRCC36-R	AGTTCAGCCAACCTCTCTGC
**Quick-Change PCR primers**	**Sequences (5′-3′)**
K12R-F	ATCTAGAGCCCTGGAgGCATCCAGGAAGTCA
K12R-R	TGACTTCCTGGATGCcTCCAGGGCTCTAGAT
K28R-F	ACAATTGCTATTGTAgAAAGTGTTGCTTTCA
K28R-R	TGAAAGCAACACTTTcTACAATAGCAATTGT
K41R-F	CGTGTTTCACAAGAAgAGGCTTAGGCATCTC
K41R-R	GAGATGCCTAAGCCTcTTCTTGTGAAACACG
K50R-F	TCTCCTATGGCAGGAgGAAGCGGAGACAGCG
K50R-R	CGCTGTCTCCGCTTCcTCCTGCCATAGGAGA
K51R-F	CCTATGGCAGGAAGAgGCGGAGACAGCGACG
K51R-R	CGTCGCTGTCTCCGCcTCTTCCTGCCATAGG
R52K-F	TATGGCAGGAAGAAGaaGAGACAGCGACGAAG
R52K-R	CTTCGTCGCTGTCTCttCTTCTTCCTGCCATA

R53K-F	GCAGGAAGAAGCGGAaACAGCGACGAAGAGC
R53K-R	GCTCTTCGTCGCTGTtTCCGCTTCTTCCTGC
K71R-F	AAGCTTCTCTATCAAgGCAACCCGCCTCCCA
K71R-R	TGGGAGGCGGGTTGCcTTGATAGAGAAGCTT
**ChIP primers (Chen et al., 2014)**	**Sequences (5′-3′)**
2D10-A-Forward	GGGCTAATTCACTCCCAACGA
2D10-A-Reverse	GGAAGTAGCCTTGTGTGTGGTAGA
2D10-B-Forward	AGCTTGCTACAAGGGACTTTCC
2D10-B-Reverse	ACCCAGTACAAGCAAAAAGCAG
2D10-C-Forward	CTGGGAGCTCTCTGGCTAACTA
2D10-C-Reverse	TTACCAGAGTCACACAACAGACG
2D10-D-Forward	GACTGGTGAGTACGCCAAAAAT
2D10-D-Reverse	TTTCCCATCGCGATCTAATTC
2D10-E-Forward	AGCAACCCTCTATTGTGTGCAT
2D10-E-Reverse	TGCGGTGGTCTTACTTTTGTTT
2D10-F-Forward	GCAGTGGGAATAGGAGCTTTGT
2D10-F-Reverse	GCTGCGCCCATAGTGCTT
2D10-G-Forward	GTCCGCCCTGAGCAAAGA
2D10-G-Reverse	TCCAGCAGGACCATGTGATC
2D10-H-Forward	ATGGCGGTCTTCCTAATGAC
2D10-H-Reverse	AACTTGTTGGCAGTCCATCC

Supplementary references:

Brasier A.R., Tate J.E., Habener J.F. (1989). Optimized use of the firefly luciferase assay as a reporter gene in mammalian cell lines. *BioTechniques*, 7, 1116-1122.

Chen, Y., Zhang, L., Estarás, C., Choi, S.H., Moreno, L., Karn, J., Moresco, J.J., Yates, J.R. and Jones, K.A. (2014). A gene-specific role for the Ssu72 RNAPII CTD phosphatase in HIV-1 Tat transactivation. *Genes & development*, 28, pp.2261-2275.

Day, J.R., Martínez, L.E., Sásik, R., Hitchin, D.L., Dueck, M.E., Richman, D.D., and Guatelli, J.C. (2006). A computer-based, image-analysis method to quantify HIV-1 infection in a single-cell infectious center assay. *J. Virol Methods*, 137, 125-133.
